# Drug-coated balloons versus drug-eluting stents in patients with acute myocardial infarction undergoing percutaneous coronary intervention: an updated meta-analysis with trial sequential analysis

**DOI:** 10.1186/s12872-023-03633-w

**Published:** 2023-12-08

**Authors:** Ahmed Abdelaziz, Abdelrahman Hafez, Karim Atta, Hanaa Elsayed, Mohamed Abdelaziz, Ahmed Elaraby, Hallas Kadhim, Ahmed Mechi, Mahmoud Ezzat, Ahmed Fadel, Ahmed Nasr, Ali Bakr, Hazem S. Ghaith

**Affiliations:** 1 Medical Research Group of Egypt (MRGE), Cairo, Egypt; 2 Faculty of Medicine, Al-Azhar University, Cairo, Egypt; 3 Institute of Medicine, National Research Mordovia State University, Saransk, Russia; 4 Faculty of Medicine, Zagazig University, Zagazig, Egypt; 5 Al Muthanna University College of Medicine, Samawah, Iraq; 6 Medicine College, Internal Medicine Department, University of Kufa, Najaf, Iraq; 7 Faculty of Medicine, Menoufia University, Menoufia, Egypt; 8 Faculty of Medicine, Sohag University, Sohag, Egypt; 9 Faculty of Medicine, Al-Azhar University, Damietta, Egypt

**Keywords:** AMI, PCI, DCB, DES, TSA, Meta-analysis

## Abstract

**Background:**

Drug-coated balloons (DCBs) are an established strategy for coronary artery disease. However, the new generation drug-eluting stent (DES) is recommended for patients with Acute myocardial infarction (AMI) for coronary artery revascularization. Our aim is to provide a comprehensive appraisal of the efficacy of DCBs in patients with AMI undergoing PCI.

**Methods:**

We searched the WOS, PubMed, Scopus, and Cochrane CENTRAL till March 2023, for studies that compared DCBs versus DES in patients with AMI undergoing PCI. We used a random-effects model to compare major adverse cardiac events (MACE), cardiac death, all-cause death, myocardial infarction, target lesion revascularization (TLR), stent thrombosis, Late lumen Loss (LLL), and minimum lumen diameter (MLD) between the two groups.

**Results:**

Thirteen studies comprising 2644 patients were included. The pooled OR showed non-inferiority of DCB over DES in terms of MACE (OR = 0.89, 95% CI [0.57 to 1.40], *p* = 0.63). When we defined MACE as a composite of cardiac death, MI, and TLR; the pooled OR favored DCB over DES (OR = 0.50, 95% CI [0.28 to 0.9], *p* = 0.02). Moreover, DCB was not inferior to DES in terms of all-cause mortality (OR = 0.88, 95% CI: 0.43 to 1.8, *p* = 0.73), cardiac mortality, (OR = 0.59, 95% CI: 0.22 to 1.56, *p* = 0.29), MI (OR = 0.88, 95% CI: 0.34 to 2.29, *p* = 0.79), stent thrombosis (OR = 1.21, 95% CI: 0.35 to 4.23, *p* = 0.76), TLR (OR = 0.9, 95% CI: 0.43 to 1.93, *p* = 0.8), LLL (MD = -0.6, 95% CI: -0.3 to 0.19, *p* = 0.64), or MLD (MD = -0.4, 95% CI: -0.33 to 0.25, *p* = 0.76).

**Conclusion:**

Our meta-analysis indicated that DCB intervention was not inferior to DES in the PCI setting in patients with AMI, and can be recommended as a feasible strategy in AMI.

**PROSPERO registration:**

CRD42023412757.

**Supplementary Information:**

The online version contains supplementary material available at 10.1186/s12872-023-03633-w.

## Introduction

Ischemic heart disease is the leading cause of mortality worldwide, and its prevalence is rising; it now accounts for nearly 1.8 million annual deaths [1]. A frequent cardiac emergency with the potential for significant morbidity and mortality is acute myocardial infarction (AMI), which can occur with or without ST segment elevation (STEMI or non-STEMI) [2]. The clinical definition of myocardial infarction indicates the presence of acute myocardial injury detected by abnormal cardiac biomarkers in a clinical setting consistent with acute myocardial ischemia [3]. The primary treatment for AMI is early myocardial reperfusion achieved through medication, surgery, or intervention [4]. Acute myocardial infarction (AMI) patients are among the patients having percutaneous coronary intervention who are at the highest risk [5].

The new generation drug-eluting stent (DES) is recommended for the treatment of patients with AMI in the 2021 ACC/AHA/SCAI guideline for coronary artery revascularization because it lowers the incidence of target vessel revascularization and stent thrombosis compared to bare-metal stent (BMS) [6]. However, the use of permanent vascular implants following the placement of DES may increase the possibility of late and very late stent thrombosis [7]. Several years following stenting, stent-associated complications like in-stent restenosis and recurrent myocardial infarction (MI) may also manifest. Dual antiplatelet therapy (DAPT) bleeding complications after stenting should not be disregarded, and stenting may not decrease mortality, or the incidence of MI recurrence compared with balloon angioplasty alone [8].

A beneficial therapeutic option to percutaneous coronary intervention (PCI) is a drug-coated balloon (DCB), a novel treatment strategy that has emerged in recent years which prevents stent thrombosis, reduces the need for dual antiplatelet therapy, and lowers the rate of restenosis by leaving no metal behind [9]. The advantage of DCB is that it could rapidly provide a homogeneous distribution and high concentration of anti-restenotic drugs into the target lesion of the culprit coronary artery without using durable polymers and stent structures [10]. They can be used in combination with bare metal stent (BMS) or alone (DCB only).

Small artery disease and in-stent restenosis have both been effectively treated using a DCB-only approach [11, 12]. Many clinical trials have also demonstrated its value in bifurcation lesions, diffuse disease, chronic total occlusions, high blood risk conditions, calcified complex lesions, and even in de novo large vessel disease [13–15]. Although some recent clinical trials have evaluated the feasibility of DCB for the treatment of AMI patients [10, 16], these individual studies do not provide very strong evidence of the exact efficacy of DCB for AMI. The effects of DCB in the treatment of AMI are still less well known. Therefore, we performed a meta-analysis to assess the clinical efficacy of DCB in the management of patients with AMI.

## Methods

We adhered to the guidelines of the Preferred Reporting Items for Systematic Reviews and Meta-Analysis (PRISMA) statement in conducting this systematic review and meta-analysis [17]. The methods employed were in strict compliance with the Cochrane Handbook of Systematic Reviews and Meta-Analysis of Interventions (version 5.1.0).

### Eligibility criteria

We included all the studies satisfying the following criteria:

1. *Population:* patients with AMI "STEMI or NSETMI".
2. *Intervention:* drug-coated balloon.
3. *Comparator:* drug-eluting stent.
4. *Outcome:* major adverse cardiac events (MACE), all-cause death, target lesion revascularization (TLR), myocardial infarction (MI), stent thrombosis, cardiac death as, late lumen loss (LLL), and mean lumen diameter (MLD).
5. *Study design:* Randomized controlled- trials (RCTs) and observational studies.

We excluded studies that were not in the English language, animal studies, and conference abstracts.

### Literature search

An extensive literature search was done on four databases (PubMed, Scopus, Web of Science, and Cochrane Library) from inception until March 30, 2023, using the following search terms: (drug-coated balloon OR DCBs) AND (myocardial infarction OR Acute coronary syndrome OR Acute myocardial infarction OR NSTEMI OR STEMI OR Non-ST-Elevation Myocardial Infarction OR ST-Elevation Myocardial Infarction) AND (Drug-Eluting Stent OR DES OR stent) AND (PCI OR Percutaneous Coronary Intervention). EndNote was used to remove duplicates. In addition, manual backward citation analysis was performed for all the references of the included studies.

### Screening of the literature search results

The literature search results were screened in a two-step process. Initially, the titles and abstracts of all articles were assessed for eligibility. Subsequently, full-text screening was conducted for the studies that met the eligibility criteria.

### Data extraction

Data from the included studies was extracted and recorded in a standardized data extraction sheet. The extracted data encompassed four main categories: (1) Characteristics of the included studies, (2) Characteristics of the study population, (3) Risk of bias domains, and (4) Outcome measures, which included MACE, all-cause death, TLR, MI, stent thrombosis, cardiac death, LLL, and MLD.

### Synthesis of results

For outcomes that involved dichotomous data, the frequency of events and the total number of patients in each group were combined to calculate the OR using the DerSimonian-Laird random-effect model. For outcomes that involved continuous data, such as mean difference (MD) and standard deviation (SD), were combined using the DerSimonian-Laird random-effect model. In cases where studies reported data at multiple time points, the last endpoint was considered for the primary analysis. All statistical analyses were conducted using Stata MP version 17 for Mac.

### Heterogeneity assessment

The Chi-square test (Cochrane Q test) was used to assess heterogeneity among studies. The Chi-square was used to calculate the I-squared according to the equation: I^2^ = Q-dfQx100%. A Chi-square *P* value less than 0.1 was considered significant heterogeneity and I-square values ≥ 50% were indicative of high heterogeneity.

### Quality assessment

Three authors evaluated the quality of the included studies independently using the Cochrane Risk of Bias 2 tool for RCTs, which involves assessing five domains: randomization process, deviation from intended interventions, outcome measurement, missing outcome data, and selection of reported results. Observational studies were evaluated using the Newcastle–Ottawa Scale (NOS) which involves three domains (selection, comparability, and outcome).

The authors' assessment decisions were classified as 'Low risk of bias', 'High risk of bias', or 'Some concerns'. Any discrepancies among the three authors were resolved through discussion with a fourth author.

To investigate the possibility of publication bias, a funnel plot that shows the relationship between effect size and standard error was created. In addition, two statistical methods were used to determine evidence of publication bias: 1) Egger's regression test and 2) the Begg and Mazumdar rank correlation test (Kendall's tau).

### Certainty assessment

We performed a certainty assessment using sensitivity analysis, also known as leave-one-out meta-analysis, to evaluate the robustness of the evidence. For each outcome included in the meta-analysis, we conducted sensitivity analysis in various scenarios by excluding one study at a time, to ensure that the overall effect size was not heavily influenced by any single study.

Due to the cumulative pooling of different trials, along with the limited amount of reported data; there is an increased risk of type 1 and 2 errors. However, a trial sequential analysis (TSA) was performed to assess whether the evidence generated from the analysis was reliable and conclusive.

The level of confidence is conclusive and sufficient, indicating no other studies are need, when the z-line of TSA curve crosses both the conventional boundary and the sequence monitoring boundary. On the other hand, if the z-line does not cross any boundary on the curve, the evidence is not conclusive, and further trials are still required. In this meta-analysis, we used an alpha error of 0.05, a beta error of 80% power, and a risk reduction of 20%.

## Results

### Literature search

**Fig. 1:**
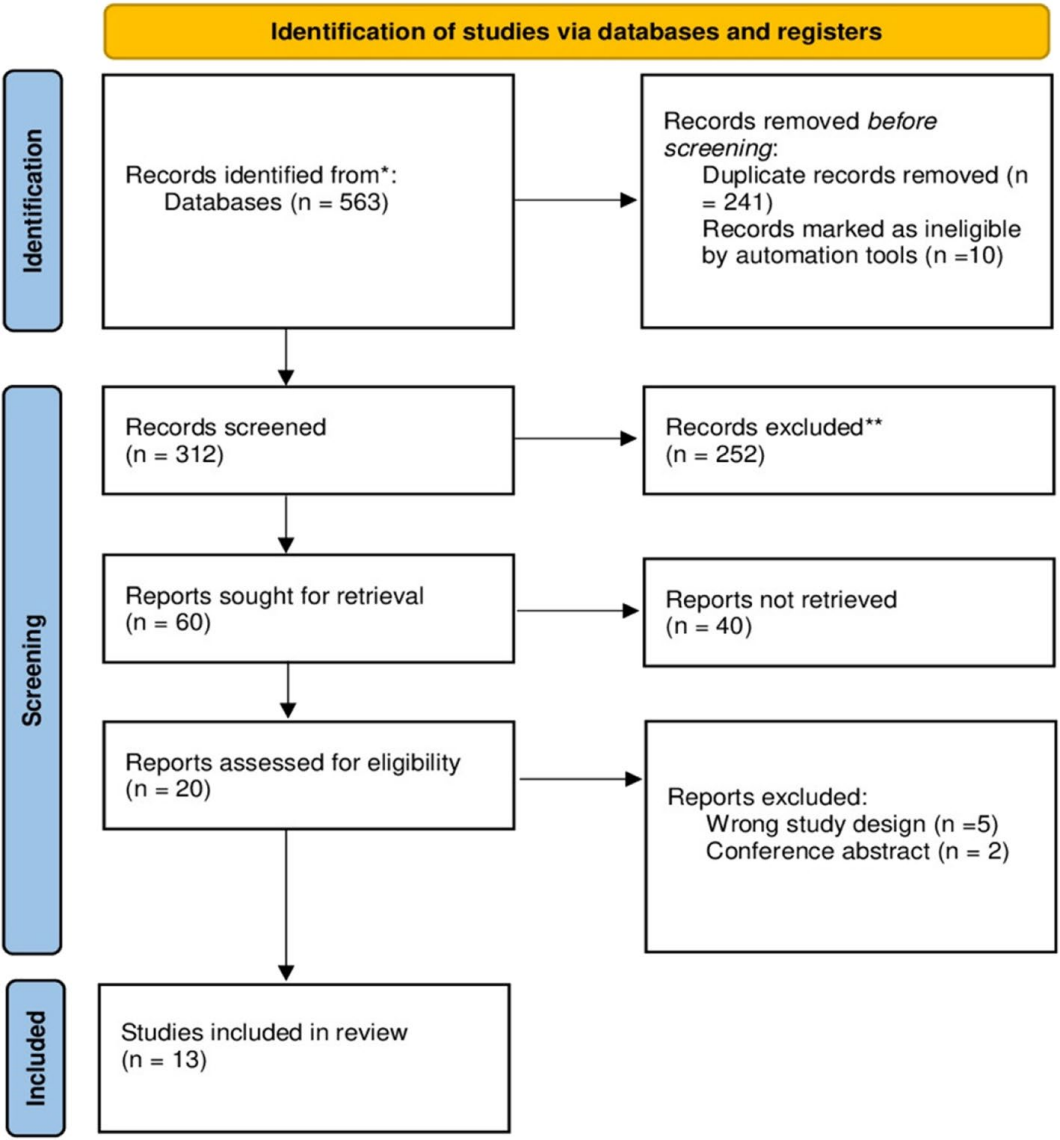
PRISMA flow diagram

Searching databases retrieved 563 results. After duplicate removal, we had 312 for screening. After titles and abstract screening, 60 articles were eligible for full-text screening. From these 60 studies, 13 studies (10 RCTs and 3 observational) were included in the meta-analysis. Also, backward citation analysis was manually done, and no further articles were included. The PRISMA flow diagram for the selection process is shown in Fig. 1.

### Characteristics of included studies

Our meta-analysis included 13 studies of 2644 patients comparing DCB with DES [10, 16, 18–28]. All included studies assessed our primary outcome, MACE. The baseline and summary of our included studies are shown in Table 1.

**Table 1:** Baseline and summary characteristics of included studies

Characteristics of All included Studies																	
**Author, year**	Type of Study	Country	Patients (n) (DCB/stent)	Type of patients	BMI (kg/m2) (DCB/stent)	Age (mean) (DCB/stent)	male, n (DCB/stent)	mean follow up (days)	Outcomes reported	Premedication	Lesion preparation	DCB type	Stent type	in-stent restenosis, %	de lovo lesions, n	small vessel lesions, n	large vessel lesions, n
**Scheller 2020** [21]	RCT	Germany	104\106	NSTEMI	28.7\28.4	66\67	69\72	276	MACE/stent thrombosis//TLR/MI/CD	aspirin plus clopidogrel, ticagrelor or prasugrel	Balloon dilation	paclitaxel iopromide-coated DCB	Paclitaxel	exclusion criteria	Inclusion citeria but no data		exclusion criteria
**Vos 2019** [29]	RCT	The Netherlands	60\60	STEMI	26.7\27.4	57.4\57.3	52\52	270	MACE/CD/ MI/TLR	aspirin plus ticagrelor or prasugrel	Thrombus aspiration and/ or balloon dilation	Paclitaxel	Sirolimus or Everolimus(Orsiro, Biotronik; or Xience, Abbott, Abbott Park, Illinois)	exclusion criteria	60/60	NA	NA
**Wang 2022** [16]	RCT	China	92\92	STEMI	N.A	49.2\49.6	88\84	365	MACE/CD/ MI/TLR/MLD/LLL	Aspirin/Clopidogrel/ β-blocker/Calcium channel blocker/RAS inhibitor/ Statin/Ezetimib/Nitrate	Thrombus aspiration and/ or balloon dilation	Paclitaxel	sirolimus	exclusion criteria	Inclusion citeria but no data		NA
**Yang 2023** [10]	Observtional	China	67\209	STEMI	27.9\27.7	38.7\39.4	66\196	365	MACE/CD/ MI/TLR	Aspirin/Clopidogrel/Ticagrelor/β-blocker/Calcium channel blocker/RAS inhibitor/ Statin/Ezetimib/Nitrate	Thrombus aspiration and/ or balloon dilation	N.A	N.A	exclusion criteria	NA	NA	NA
**Belkacemi 2012** [27]	RCT	the Netherlands/Italy	50\49\51	STEMI	N.A	59.7\59.9	41\41\42	180	MACE/ LLL	acetylsalicylic acid/clopidogrel/Heparin/glycoprotein IIb/IIIa inhibitors	Thrombus aspiration and/ or balloon dilation	DIORVR coronary angio- plasty balloon	paclitaxel DES	10\9	50/49	NA	NA
**Besic 2015** [15]	RCT	Croatia	41\44	NSTEMI	29\28.1	63\68	33\35	180	MACE/LLL/TLR/ST	clopidogrel/aspirin/unfractionated heparin/glycoproglycoprotein (GP) IIb/IIIa inhibitors (eptifibatide)	Thrombus aspiration and/ or balloon dilation	Elutax and SeQuent Please (B. Braun AG, Melsungen, Germany)	new cobalt chromium stent (Genius Magic stent, Eurocor)	exclusion criteria	41/44	NA	NA
**García-Touchard 2017** [24]	RCT	Spain	110\112	STEMI	27.7\27.5	60\62	94\96	365	MACE/CD/TLR/MLD/LLL	aspirin/clopidogrel/prasugrel/ticagrelor/Heparin/glycoprotein IIb/IIIa inhibitors	Thrombus aspiration and/ or balloon dilation	paclitaxel-eluting balloon	new cobalt chromium stent (Genius Magic stent, Eurocor)	exclusion criteria	NA	NA	NA
**Gobić 2017**	RCT	Croatia	38\37	STEMI	29.4\28.2	57\45	71\73	180	CD/MI/TLR/ST/LLL	acetylsalicylic acid/clopidogrel/unfractionated heparin	thrombus aspiration or balloon dilation	Sequent Please (B.Braun,Melsungen, Germany)	Sirolimus (Biomime, Meril Life Sciences, Vapi, India)	NA	Inclusion citeria but no data		exclusion criteria
**Hao 2021**	RCT	China	38\42	STEMI	26\25	59\56	30\35	365	MACE/ LLL/TLR	enteric-coated aspirin tablets/ clopidogrel/ atorvastatin calcium/ anti-hypertensive and blood sugar control treatments for underlying diseases	thrombus aspiration and balloon expansion	N.A	N.A	exclusion criteria	NA	NA	NA
**Merinopoulos 2023** [28]	Observtional	UK	452/687	STEMI	NA	66/66	330/509	365	MACE/mortality/stent thrombosis/TLR	acetylsalicylic acid/clopidogrel/Heparin/glycoprotein IIb/IIIa inhibitors	Thrombus aspiration and/ or balloon dilation	paclitaxel-eluting balloon	Sirolimus (Biomime, Meril Life Sciences, Vapi, India)	exclusion criteria	452/687	NA	Inclusion citeria but no data
**Wang 2020** [19]	RCT	China	38/42	STEMI	26/25	59/56	79/83	365	CD, MI, TLR, LLL	aspirin plus clopidogrel	Thrombus aspiration and/ or balloon dilation	Sequent Please	DES	NA	NA	NA	NA
**Liu 2020** [18]	RCT	China	33/32	STEMI	NA	NA	NA	365	CD, MI, TLR, ST, 12 LLL	aspirin plus ticagrelor	Balloon dilation	Sequent Please	Xience V DES	exclusion criteria	Inclusion citeria but no data		NA

Characteristics of All included Studies																
**Author, year**	upfront hybrid PCI (DCB + DES), %	Bailout stenting rate in the DCB (%)	DAPT (months) (DCB/ stent)	Medical Conditions, n (DCB/ stent)							Angiographic assessment, (DCB/ stent)					
				History of stroke	History of MI	DM	HTN	Hyperlipidaemia	Current smoker	PAD	LAD, n	LCx, n	RCA, n	Number of stents implanted, mean	Stent diameter, mean	stent length, mean
**Nijhoff 2015** [15]	NA	10%	N.A	N.A	2\3	5\2	14/15	7\16	21\28	N.A	18/18	NA	13/20	NA	NA	NA
**Scheller 2020** [21]	15%	15%	12\12	6\9	20\17	28/38	82\93	52\48	35\43	9\7	51/46	40/44	32/30	NA	2.6/2.5	18.6/17.4
**Vos 2019** [29]	NA	18%	12\12	N.A	N.A	8\4	18\19	10\8	28\24	1\0	19/24	NA	29/28	NA	NA	NA
**Wang 2022** [16]	NA	9.50%	3\12	N.A	92\92	71\79	67\65	61\58	N.A	N.A	64/64	24/19	29/30	1.27/1.23	2.69/2.7	16.92/16.97
**Yang 2023** [10	exclusion criteria	NA	6\12	3\8	67\209	15\36	31\95	18\44	53\173	N.A	19/96	29/35	19/78	1\2	2.57/3	26/30
**Belkacemi 2012** [27]	NA	NA	12\12	N.A	1\2\0	3\2\6	17\15\18	13\16\11	19\28\29	N.A	24/18	13\11	13/20	1.3/1.2	2.98/2.88	24.4/25.4
**Besic 2015** [15]	NA	NA	12\12	N.A	4\6	13\12	41\44	36\39	18\9	N.A	22/16	2\6	14/15	1\1	3\3	18/18
**García-Touchard 2017** [24]	NA	NA	12\12	N.A	3\5	15\22	42\55	45\57	81\81	N.A	25/36	23/20	62/56	NA	3\3	18.8/18
**Gobić 2017**	NA	7.30%	12\12	N.A	N.A	5\11	32\35	9.5\18.9	43.9\56.8	N.A	NA	NA	NA	NA	NA	NA
**Hao 2021**	NA	4%%	6\12	N.A	N.A	10\15	8\12	N.A	24\28	N.A	19/22	7\8	12\12	NA	NA	NA
**Merinopoulos 2023 **[28]	NA	5.30%	12\12	18\12	30/35	63/83	183/241	79/100	254/435	7\7	196/256	78/103	175/321	NA	NA	NA
**Wang 2020** [19]	NA	9.50%	6\12	NA	NA	NA	NA	NA	NA	NA	NA	NA	NA	NA	NA	NA
**Liu 2020** [18]	NA	5.70%	12\12	NA	NA	NA	NA	NA	NA	NA	NA	NA	NA	NA	NA	NA

### Risk of bias assessment

The ten RCTs were assessed using ROB-2 tool as following:

- Randomization process

**Fig. 2:**
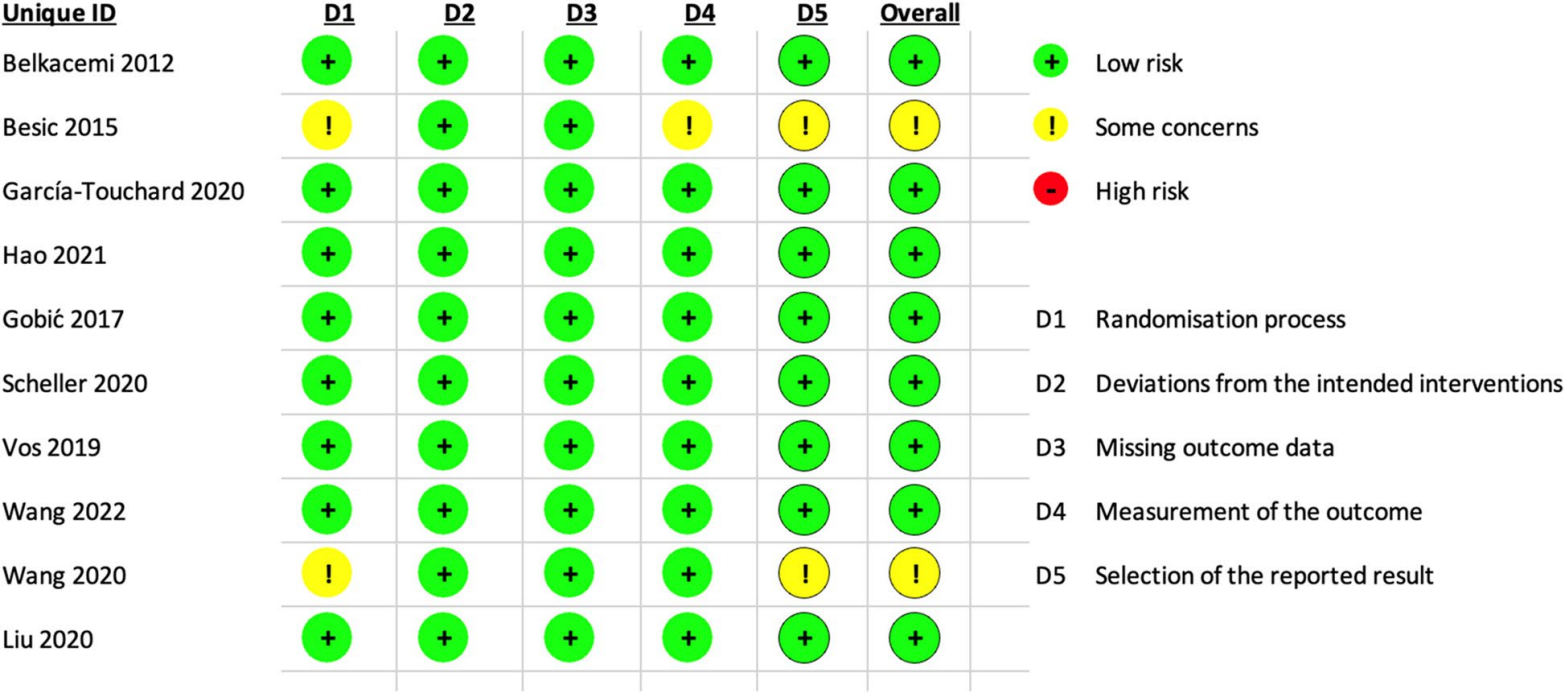
Risk of bias assessment 2 tool (ROB-2)

All the ten included trials reported a correct method of random sequence generation through computer- generated random sequence or permuted block randomization. Allocation concealment was achieved in eight studies mostly through sealed envelopes. In addition, there were no baseline differences between the intervention groups in all the studies. The risk of bias summary is shown in Fig. 2.

- Deviations from intended interventions

Double- blinding of wasn’t achievable in all the studies but this didn’t have a substantial impact on the results. Appropriate analysis method (Intention to treat analysis) was observed in almost all the studies.

- Incomplete outcome data

All the studies reported nearly complete outcome data.

- Measurement of the outcome

All the studies showed a correct outcome measurement. However, blinding of the outcome assessors was not observed in all the studies.

- Selection of the reported result

Almost all the studies reported results according to a registered protocol with appropriate selection of the reported result.

The three observational studies (Nijhoff 2015, Yang 2023, and Merinopoulos 2023) were assessed using the NOS as following:

All studies were truly representative of the patients included. The control group was selected from the same community as the exposed group in Yang 2023 but no description of the derivation of the non- exposed cohort in Nijhoff 2015. The ascertainment of exposure was confirmed by surgical records. Also, the two groups included in all studies were comparable. They also showed adequate periods of follow-up. Therefore, the overall quality of all studies is good, as shown in Table 2.

**Table 2:** NOS scale for observational studies

Cohort studies															
Baseline							**Selection**			**Comparability**	**Outcome**				**Quality Score**
Study Title	**First Author**	**Year**	**Study Design (Prospective or retrospective)**	**Follow-up period/Duration of exposure**	**Sample (n)**	**Age at baseline mean (SD)**	**Representativeness of the exposed cohort**	**Selection of the non exposed cohort**	**Ascertainment of exposure**	**Demonstration that outcome of interest was not present at start of study**	**Comparability of cohorts on the basis of the design or analysis**	**Assessment of outcome**	**Was follow-up long enough for outcomes to occur**	**Adequacy of follow up of cohorts**	
**Nijhoff 2015 **[23]	Freek Nijhoff	2015	prospective cohort	12 m	40	57.9 ± 10.0	*	*	*	*	*	*	*	*	Good quality
**Yang 2023 **[10]	Yi-Xing Yang	2023	retrospective cohort	12 m	276	39.3 ± 4.1	*	*	*	*	*		*	*	Good quality
**Merinopoulos 2023 **[28]	Merinopoulos	2023	prospective cohort	12m	1139	66 ± 12	*	*	*	*	*	*	*	*	Good quality

### Outcomes

#### MACE

**Fig. 3:**
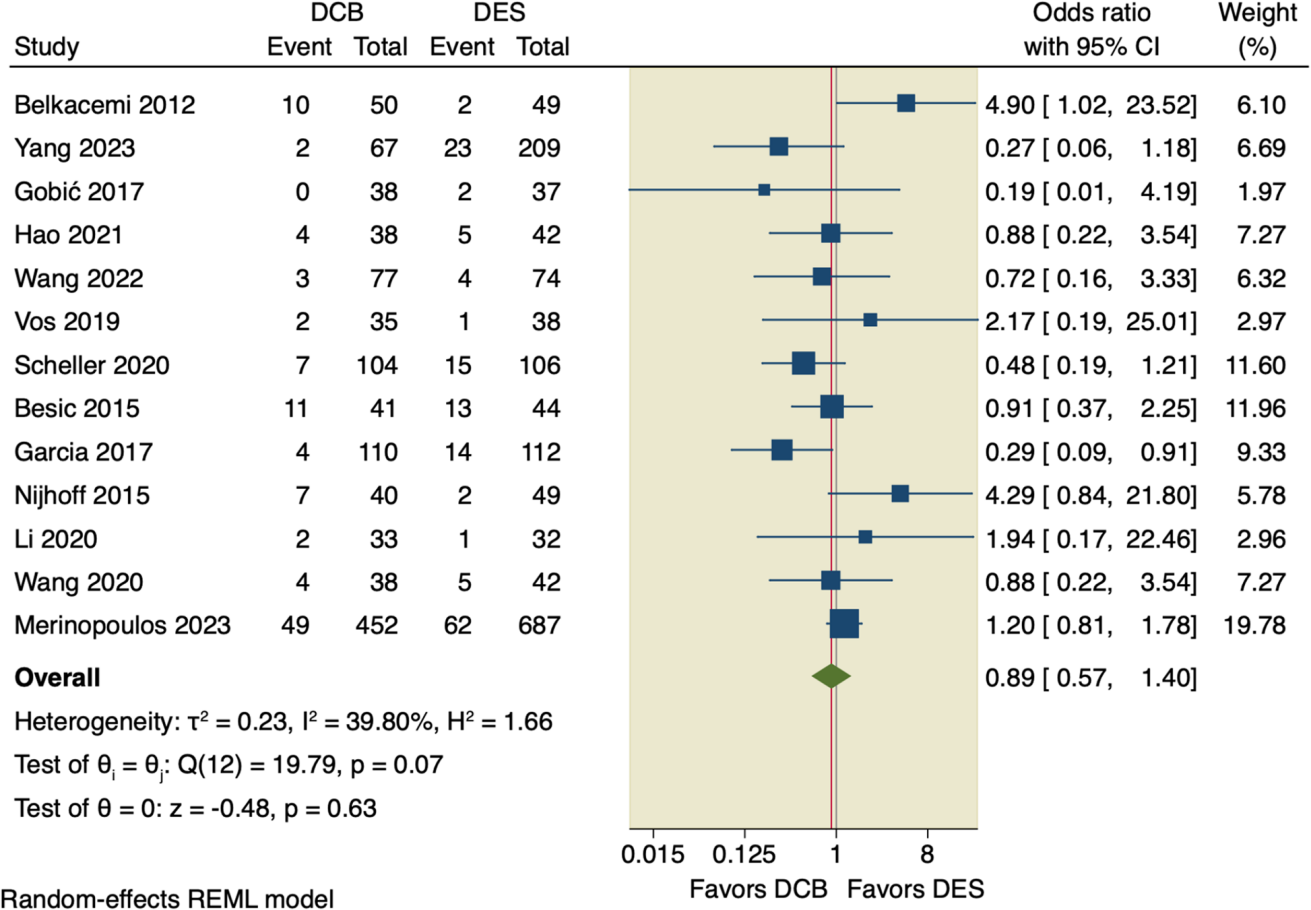
Forest plot of MACE

All included studies assessed MACE with an incidence rate of 9.35% (105 of 1123) in the DCB group, and 9.8% (149 of 1521) in the DES group. The pooled OR did not favor DCB over DES (OR = 0.89, 95% CI [0.57 to 1.4], *p* = 0.63); the pooled studies were little heterogenous (I^2^ = 39.8%, *p* = 0.07), as shown in Fig. 3.

**Fig. 4:**
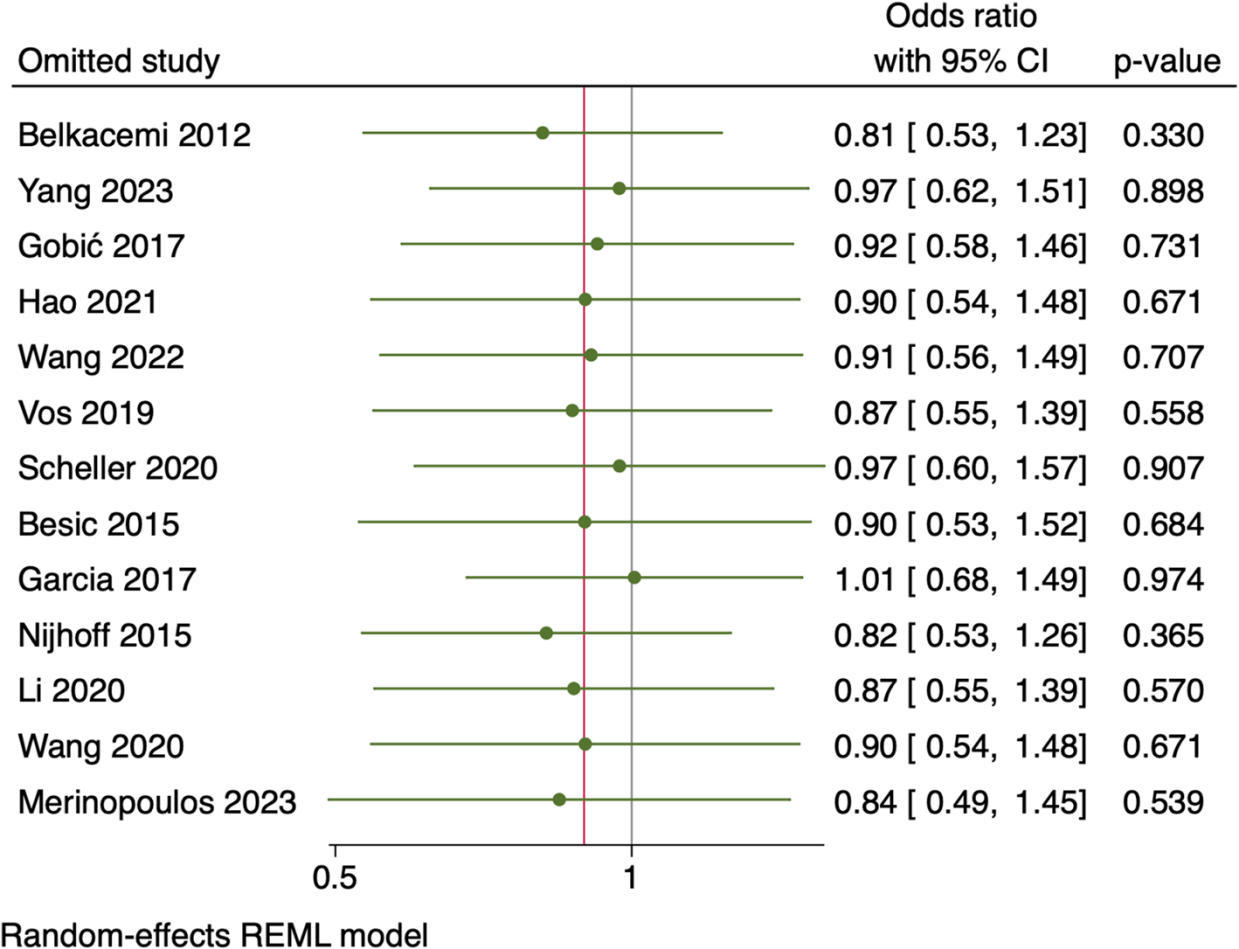
Leave-one-out analysis of MACE

Leave-one-out sensitivity analysis showed that no single study had a disproportional effect on the pooled OR, that varied from 0.81 by excluding Belkacemi et al. and by 1.01 when excluding Garcia et al., as shown in Fig. 4.

We performed a subgroup analysis based on the type of disease, as 11 of our included studies that assess MACE were on STEMI patients, while only 2 studies were on NSTEMI, in which the pooled analysis showed no significant difference between the two subgroups (OR = 0.89, 95% CI [0.35 to 1.27], *p* = 0.22 for NSTEMI studies, and OR = 0.98, 95% CI [0.55 to 1.75], *p* = 0.96, for STEMI studies). The pooled studies for the STEMI subgroup were heterogenous (I^2^ = 47.19%, *p* = 0.07), as shown in Supplementary Fig. 1.

We further investigated the clinical heterogeneity in only STEMI studies using leave-one-out sensitivity analysis model, and no single study had a disproportional effect on the overall effect estimate, as shown in Supplementary Fig. 2.

We also performed a subgroup analysis based on the indication of the intervention, as most of our studies compared DCB with DES, in which the pooled analysis showed no significant difference between all subgroups (OR = 0.92, 95% CI [0.49 to 1.75], *p* = 0.8, 0.53, 95% CI [0.18 to 1.49], *p* = 0.23, and 1.02, 95% CI [0.62 to 1.68], *p* = 0.94) for DCB plus BMS versus BMS, DCB versus BMS, and DCB versus DES, respectively, as shown in Supplementary Fig. 3.

We performed a subgroup analysis based on study type, of which the pooled analysis showed no significant difference between the two interventions (OR = 1.1, 95% CI [0.29 to 4.22], *p* = 0.89) for observational studies, and OR = 0.79, 95% CI [0.48 to 1.32], *p* = 0.37 for RCTs studies, as shown in Supplementary Fig. 4. Another sensitivity analysis was done by excluding Chinese studies, of which the pooled OR did not favor DCB over DES (OR = 0.87, 95% CI [0.52 to 1.47], *p* = 0.61); the pooled studies were moderately heterogenous (I^2^ = 50.92%, *p* = 0.03), as shown in Supplementary Fig. 5.

**Fig. 5:**
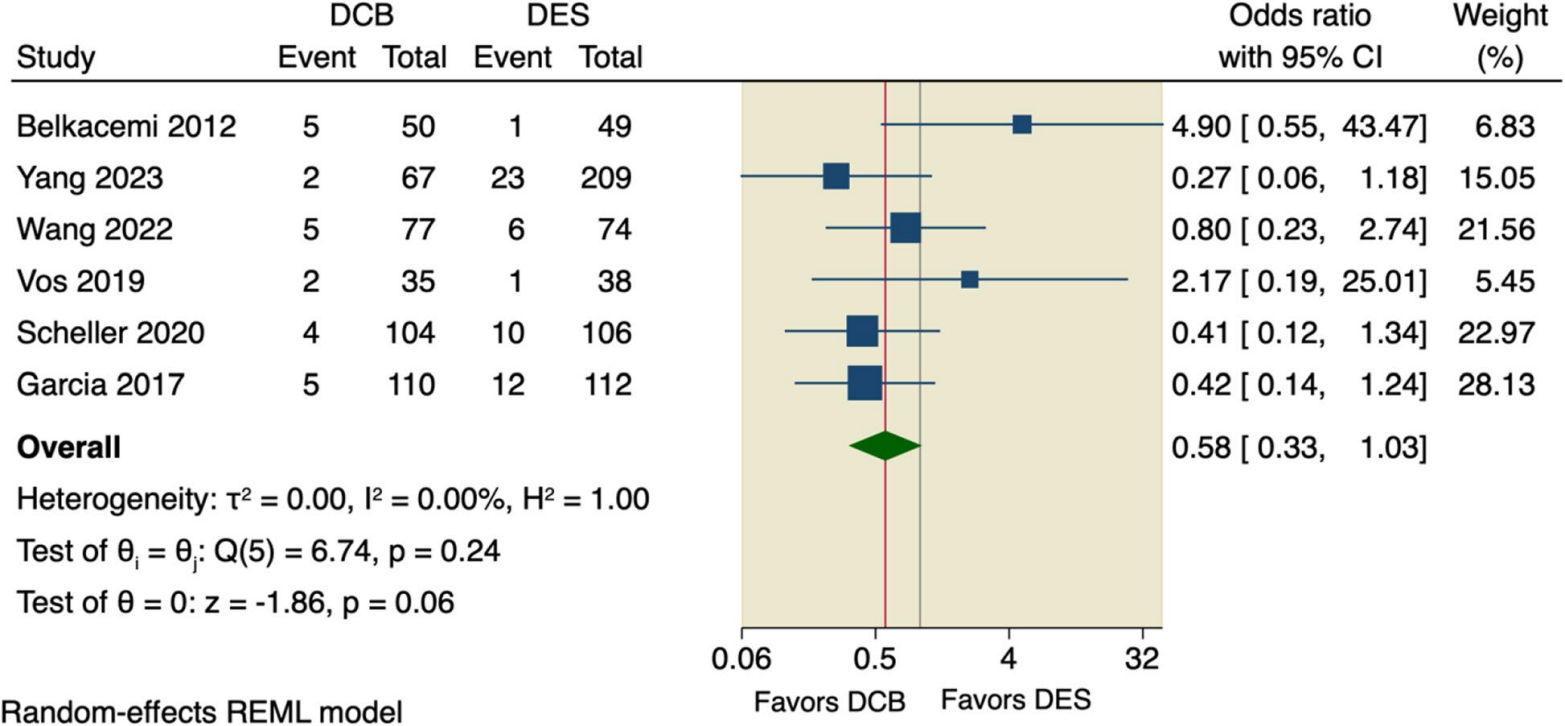
Forest plot of calculated MACE

**Fig. 6:**
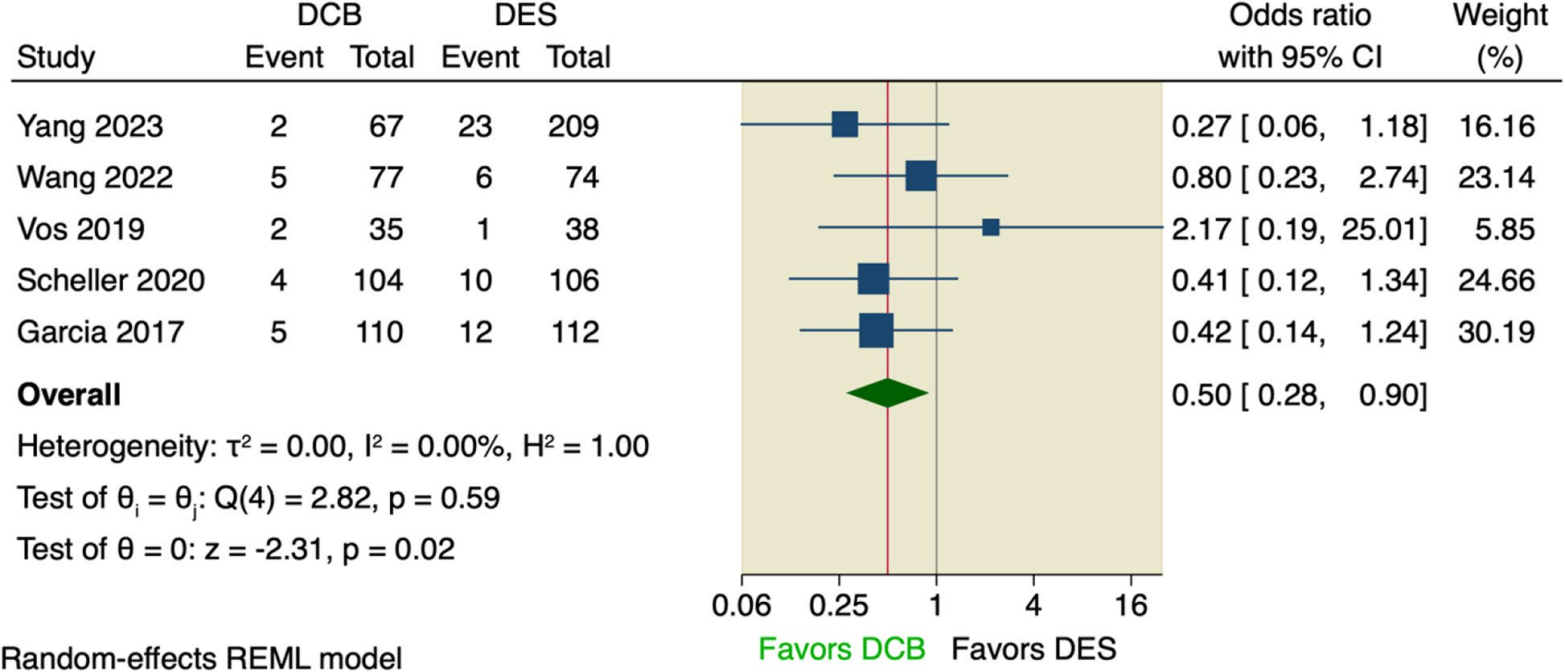
Sensitivity analysis of calculated MACE

We further pooled studies that defined MACE as a composite of only cardiac death, MI, and TLR of which the pooled analysis did not show any superior effect of DCB over DES (OR = 0.58, 95% CI [0.33 to 1.03], *p* = 0.06); the pooled studies were homogenous (I^2^ = 0.00%, *p* = 0.24), as shown in Fig. 5. And upon excluding Belkacemi et al., the pooled analysis showed that DCB lowered the incidence of MACE compared to DES (OR = 0.50, 95% CI [0.28 to 0.90], *p* = 0.02); the pooled studies were homogenous (I^2^ = 0.00%, *p* = 0.59), as shown in Fig. 6.

**Fig. 7:**
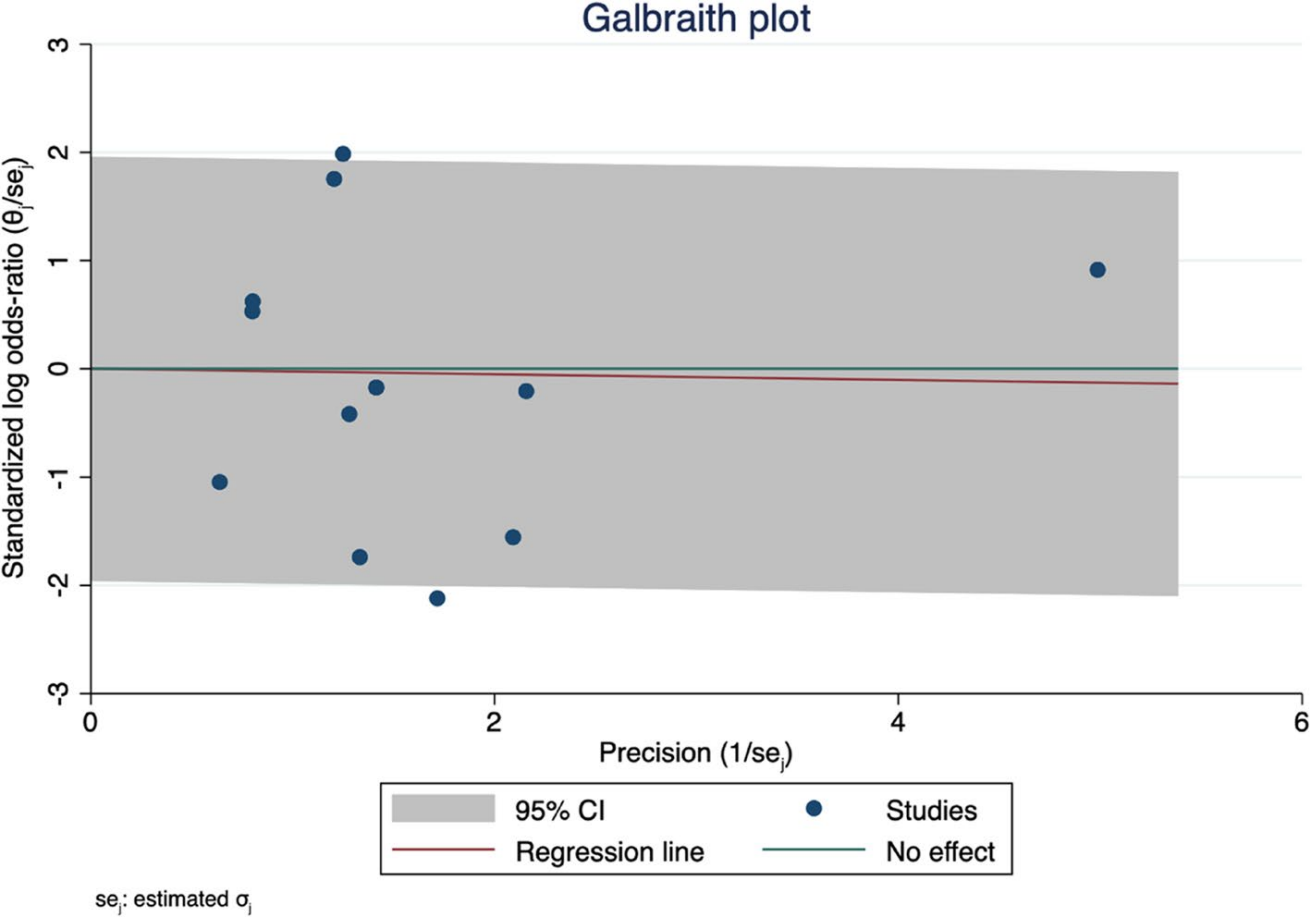
Galbraith plot MACE. The two omitted studies were Garcia 2017 and Belkacemi 2012

**Fig. 8:**
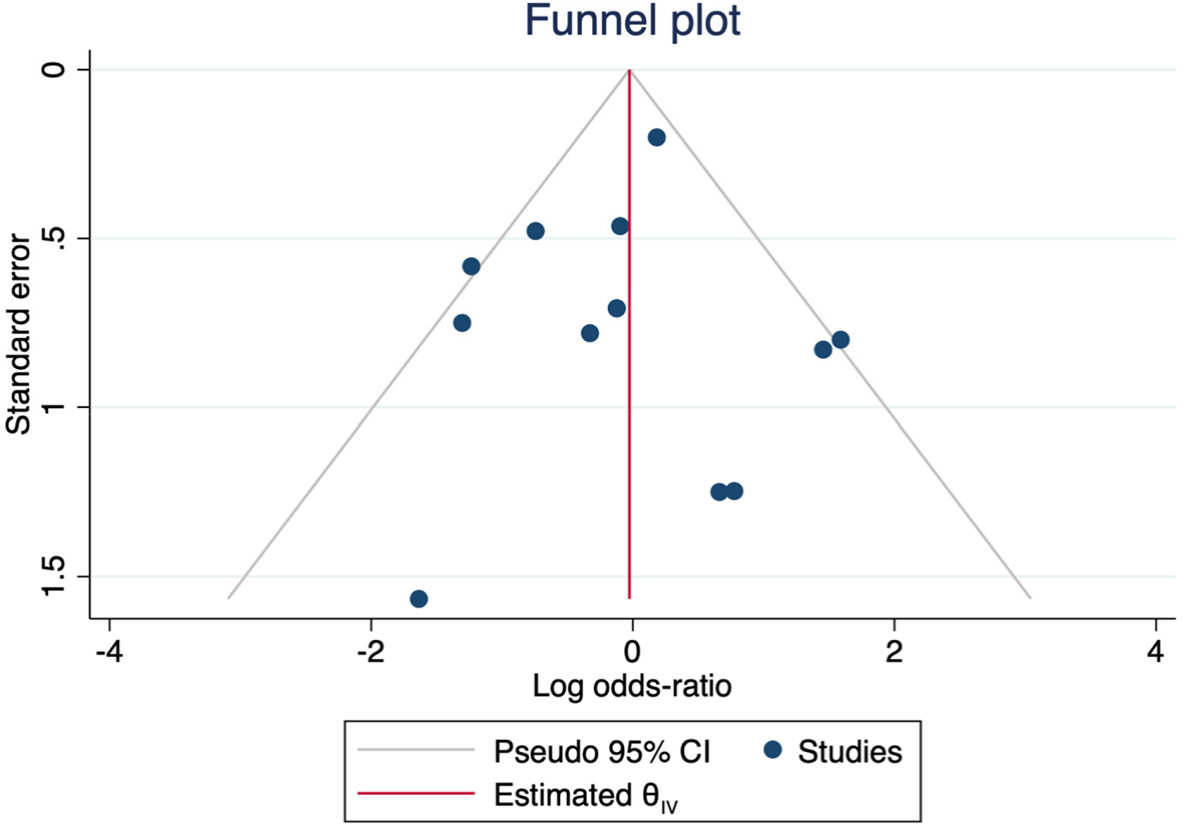
Funnel plot MACE (egger’s test = 0.07). The two omitted studies were Garcia 2017 and Belkacemi 2012

We also tested the heterogeneity using the Galbraith plot, and by inspection, there were two studies out of the 95% CI precision area, indicating their heterogeneity from other studies, as shown in Fig. 7. Moreover, we used the funnel plot to detect for any publication bias, and by inspection, we detected a slight asymmetry indicating a possible publication bias, as shown in Fig. 8.

**Fig. 9:**
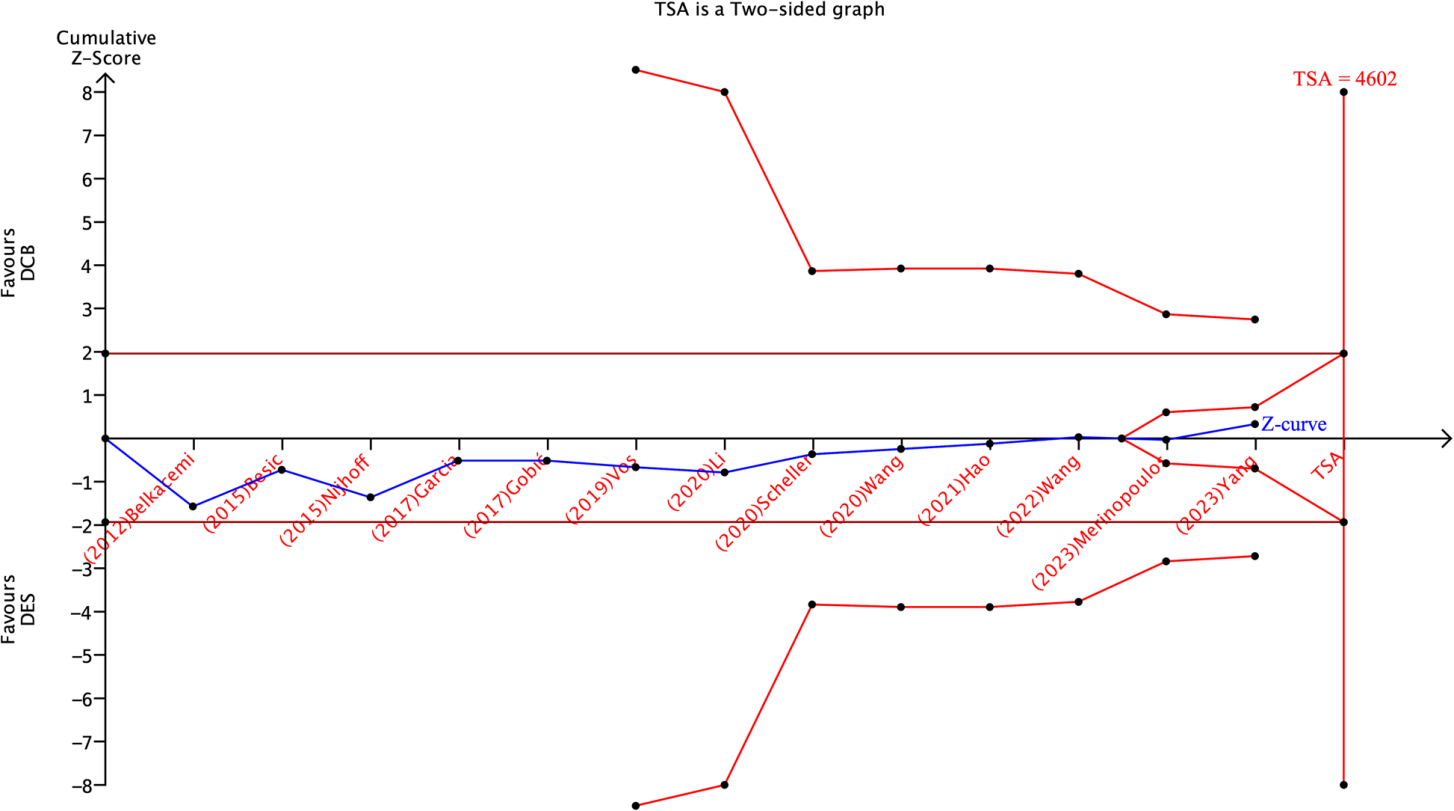
TSA report of MACE

We conducted a TSA analysis for all 13 studies assessing MACE, as shown in Fig. 9; the cumulative Z-line did not cross either the conventional boundary of the benefit or the sequential monitoring boundary in the benefit area, suggesting that our evidence did not favor DCB over the DES and further trials are needed.

#### Secondary outcomes

Our analysis did not detect any significant difference between the DCB group and the DES groups regarding all-cause mortality (OR = 0.88, 95% CI: 0.43 to 1.8), cardiac mortality, (OR = 0.59, 95% CI: 0.22 to 1.56), incidence of MI (OR = 0.88, 95% CI: 0.34 to 2.29), incidence of stent thrombosis (OR = 1.21, 95% CI: 0.35 to 4.23), or incidence of TLR (OR = 0.9, 95% CI: 0.43 to 1.93) as shown in Supplementary Figs. 6-10. The pooled studies assessing all-cause mortality, cardiac mortality, incidence of MI, or incidence of stent thrombosis were homogenous with the following values respectively: (I^2^ = 9.77%, *p* = 0.51; I^2^ = 0.00%, *p* = 1; I^2^ = 0.00%, *p* = 0.82; and I^2^ = 0.00%, *p* = 0.76). The pooled studies assessing TLR were heterogenous (I^2^ = 34.57%, *p* = 0.15). A leave-one-out sensitivity analysis was performed in which the pooled OR was decreased slightly by 0.19 by excluding Belkacemi et al. while it increased slightly by 0.19 when excluding Garcia et al. as shown in Supplementary Figs. 11.

The pooled estimate did not detect any significant difference between the DCB group and the DES group regarding LLL (MD = -0.06, 95% CI: -0.30 to 0.19) or MLD (MD = -0.04, 95% CI: -0.33 to 0.25). The pooled studies assessing LLL and MLD were heterogenous with the following values respectively: (I^2^ = 95.45%, *p* = 0.01; and I^2^ = 93.3%, *p* = 0.01) as shown in Supplementary Figs. 12-13. The leave-one-out sensitivity analysis for the two outcomes showed that no single study had a disproportional effect on the overall estimate as shown in Supplementary Figs. 14-15.

## Discussion

Our meta-analysis included 13 studies with 2644 patients comparing DCB versus DES for the treatment of acute myocardial infarction. The incidence of MACE was not significantly different between the two groups, and subgroup analyses based on disease type and intervention indication did not show significant differences either. Sensitivity analyses excluding observational and Chinese studies did not change the results. However, a pooled analysis of studies defining MACE as a composite of only cardiac death, MI, and TLR favored DCB over DES when excluding Belkacemi et al. The rationale behind the latter findings could be justified by the following: 1) The utilization of DCB in their investigation might have involved the concurrent presence of BMS, as bailout stenting was relied upon in scenarios where residual edge dissections or incomplete lesion coverage were observed; 2) The absence of pre-dilation in the DCB group and the lack of additional DCB dilation in the event of supplementary BMS implantation could have had a detrimental influence on the obtained results; 3) It should be noted that the Belkacemi et al. study. was primarily designed to assess angiographic outcomes and was not specifically intended to identify clinical disparities between the examined groups. Moreover, there were no significant differences in all-cause mortality, cardiac mortality, MI, stent thrombosis, or TLR. The pooled estimates for late lumen loss and minimum lumen diameter were not significantly different between the two studied groups.

STEMI can be effectively treated through primary percutaneous coronary intervention (pPCI), which is known to be the most efficient method of reperfusion. Research has shown that using stenting during pPCI can reduce the likelihood of repeat revascularization [1, 30]. However, stenting may also increase the risk of thrombotic complications in the long run, as well as the development of in-stent restenosis (ISR) [31, 32]. Therefore, it may be beneficial to avoid permanent implants to prevent stent-related complications in STEMI patients. An alternative approach using DCBs may be more attractive as it provides a uniform distribution of the antiproliferative drug and also reduces inflammation of the endothelial cells [33, 34]. Additionally, using DCB offers benefits such as a decreased occurrence of restenosis, a shorter period of dual antiplatelet therapy to lower the chance of bleeding, and the capacity to encourage additional recovery of endothelial function without leaving any metal objects in the blood vessels, unlike DES implantation. So, the absence of a difference in our study between DCB and DES could be considered an advantage for DCB due to the additional benefits mentioned earlier.

Megaly et al. performed a meta-analysis comparing drug-coated balloons (DCBs) to stenting in patients undergoing percutaneous coronary intervention (PCI) [5]. The study included seven randomized controlled trials with a total of 1,823 patients. The analysis showed that DCBs were not inferior to stenting in terms of overall MACE and its various components, including TLR, at a mean follow-up of 9 months after PCI. Patients with AMI are at a higher risk of stent-related events in the short and long term. The use of DCBs in these high-risk patients may provide similar protection against reintervention in the early period after PCI while avoiding the long-term complications of leaving a metallic stent in the coronary arteries [5]. However, more extensive, and longer-term studies are needed to determine any significant differences in long-term outcomes between the two strategies.

Nicola et al. were the first to conduct a study on using DCB exclusively in pPCI, which showed promising results with only 5 MACEs occurring in one year. However, half of the patients required additional stenting [29]. The DCBUT trial further demonstrated the superiority of DCB over BMS in high-bleeding risk patients with ACS, with only one patient experiencing MACEs in the DCB group [35]. Zhang meta-analysis found that DCB had comparable clinical outcomes to second-generation DES in patients with AMI, but with favorable outcomes compared to BMS. While paclitaxel is commonly used for balloon coating, there is growing evidence that sirolimus-coated balloons are safe and clinically feasible [36]. The SIRPAC study showed no significant difference in clinical endpoints between paclitaxel and sirolimus-coated balloons at 12-month follow-up [37]. Using the DCB-only strategy offers potential benefits in high thrombus load and inflammation situations, without the need for metal struts at the peak inflammatory state in STEMI, leading to better preservation of endothelial function and reduced risk of thrombosis. In contrast, DES use is associated with accelerated progression and increased prevalence of in-stent neo-atherosclerosis, leading to a higher rate of very late stent thrombosis, which can be reduced using second-generation DES but with a permanent impact on vascular structure and function [38–40].

In our study, we updated the available evidence about DCB and DES on AMI patients regarding their MACE by recruiting 13 studies including 2644 patients in our analysis. This by far is the largest meta-analysis conducted on this topic as we know. Additionally, we did a lot of stratified analyses on our patients regarding the type of MI, the indication for the intervention, and the specific definition of MACE as a composite of only cardiac death, MI, and TLR. Our latter stratification enhanced our evidence compared to previous meta-analyses as the definitions of MACE vary across studies, with some defining it as a composite of death, any myocardial infarction, and target vessel revascularization, while others define it as a combination of cardiac death, reinfarction, or revascularization of target lesions. Some studies also include stent thrombosis or stroke as part of the MACE definition. The differences in MACE definitions used in our included studies can make it challenging to compare the effectiveness of various interventions across different trials. Furthermore, leave one out analysis for our outcomes helped us to identify if there were any outliers that could affect the pooled estimates and make sure that it was not dependent on individual studies.

However, our study was not free of limitations, and they were as follows: 1) The omission of stratification for DES based on their generation or the specific drug coated on the balloon in the DCB arm might have introduced a potential bias in our results, as they are primarily dependent on their ability to release the antiproliferative drug consistently and continuously over a specific duration. Different generations or variations in the specific drug coatings could impact our study outcomes; 2) Variation of follow-up periods among our studies that could make our results vulnerable to differential attrition meaning that longer follow-up periods can lead to differential loss to follow-up between groups and time-dependent confounding; 3) NSTEMI patients were included only in two studies so the results regarding these patients should be interpreted cautiously; 4) The timing of DCB intervention could not be evaluated as to insufficient data provided in the included studies; however we recommend further studies to outline the exact timing of DCB for guiding interventional cardiologist on decision making.

## Conclusion

MACE incidence wasn't significantly different between DCB and DES groups in 13 studies, and subgroup analyses didn't reveal differences either. A pooled analysis favored DCB over DES when defining MACE as cardiac death, MI, and TLR, excluding Belkacemi et al. No significant differences were found in mortality, MI, stent thrombosis, TLR, late lumen loss, and minimum lumen diameter. Sensitivity analyses excluding observational and Chinese studies did not alter the results. Future studies should stratify DES based-on generation and type of drug coating to minimize potential biases. Standardizing follow-up periods across studies and including a larger number of NSTEMI patients in future research could also help overcome the limitations identified in our study.

### Supplementary Information

**Additional file 1.**

**Additional file 2.**

## Data Availability

The data underlying this article are available in the article and in its online supplementary material.
