# Correction: Drug-coated balloons versus drug-eluting stents in patients with acute myocardial infarction undergoing percutaneous coronary intervention: an updated meta-analysis with trial sequential analysis

**DOI:** 10.1186/s12872-023-03688-9

**Published:** 2024-01-08

**Authors:** Ahmed Abdelaziz, Abdelrahman Hafez, Karim Atta, Hanaa Elsayed, Mohamed Abdelaziz, Ahmed Elaraby, Hallas Kadhim, Ahmed Mechi, Mahmoud Ezzat, Ahmed Fadel, Ahmed Nasr, Ali Bakr, Hazem S. Ghaith

**Affiliations:** 1 Medical Research Group of Egypt (MRGE), Cairo, Egypt; 2 Faculty of Medicine, Al-Azhar University, Cairo, Egypt; 3 Institute of Medicine, National Research Mordovia State University, Saransk, Russia; 4 Faculty of Medicine, Zagazig University, Zagazig, Egypt; 5 Al Muthanna University College of Medicine, Samawah, Iraq; 6 Medicine College, Internal Medicine Department, University of Kufa, Najaf, Iraq; 7 Faculty of Medicine, Menoufia University, Menoufia, Egypt; 8 Faculty of Medicine, Sohag University, Sohag, Egypt; 9 Faculty of Medicine, Al-Azhar University, Damietta, Egypt

**Correction: BMC Cardiovascular Disorders (2023) 23:605**

10.1186/s12872-023-03633-w

Following publication of the original article [1], the affiliation detail for the Abdelrahman Hafez were incorrectly given as “Institute of Medicine, National Research Mordovia State University, Saransk, Russia.” but should have been “Faculty of Medicine, Al-Azhar University, Cairo, Egypt”.

The original article has been corrected.

## References

[1] Abdelaziz A, Hafez A, Atta K (2023). Drug-coated balloons versus drug-eluting stents in patients with acute Myocardial Infarction undergoing percutaneous coronary intervention: an updated meta-analysis with trial sequential analysis. BMC Cardiovasc Disord.

