# Supplementary material for: Drug-coated balloons versus drug-eluting stents in patients with acute myocardial infarction undergoing percutaneous coronary intervention: an updated meta-analysis with trial sequential analysis
Source: BMC Cardiovasc Disord. 2023 Dec 8;23:605. doi: 10.1186/s12872-023-03633-w (PMC10709955; doi:10.1186/s12872-023-03633-w)
Supplement: Supplementary file 1 — Additional file 1. [file 12872_2023_3633_MOESM1_ESM.docx]

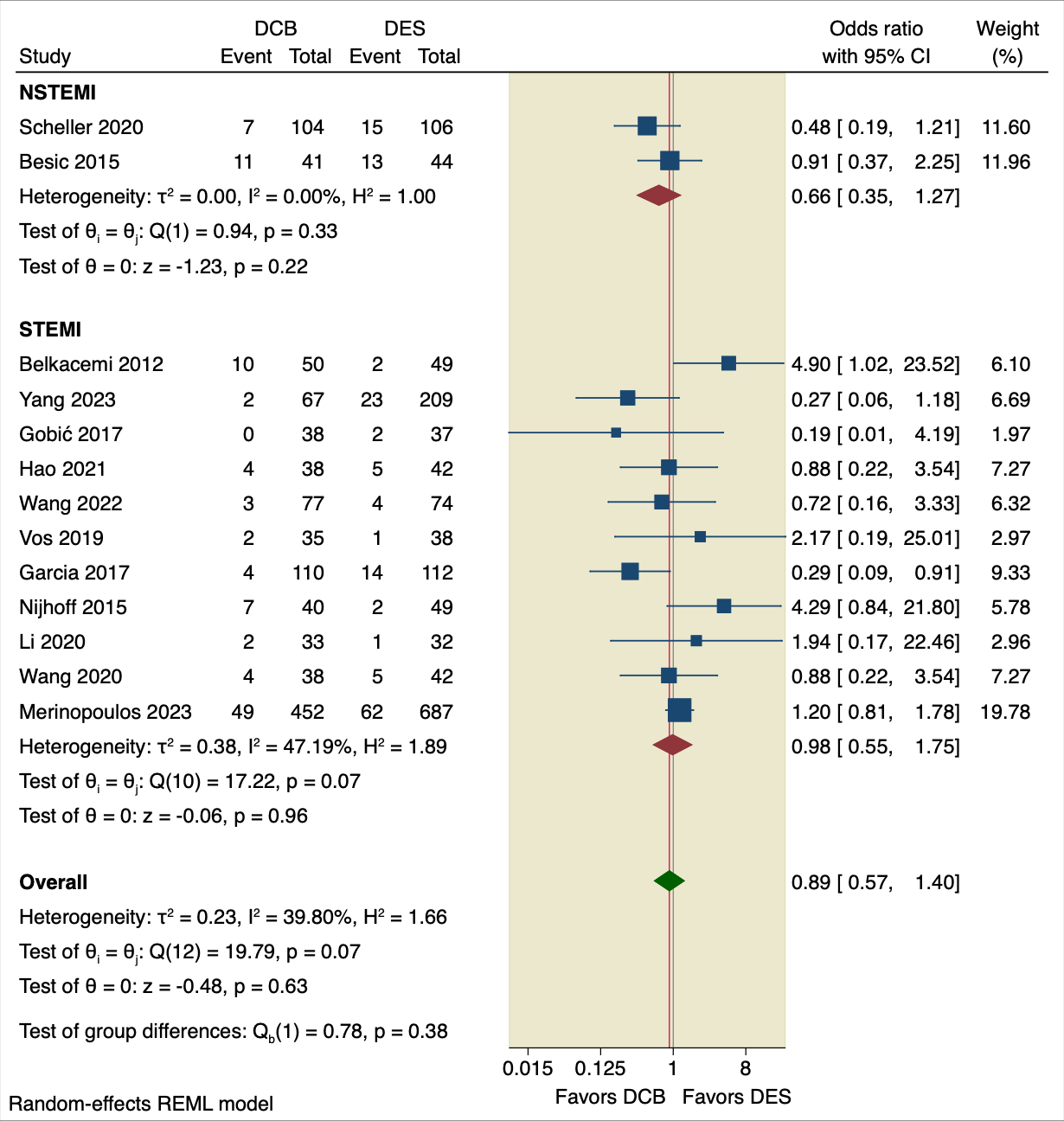


**Supplementary Figure 1:** Subgroup analysis of MACE based on the type of the disease.


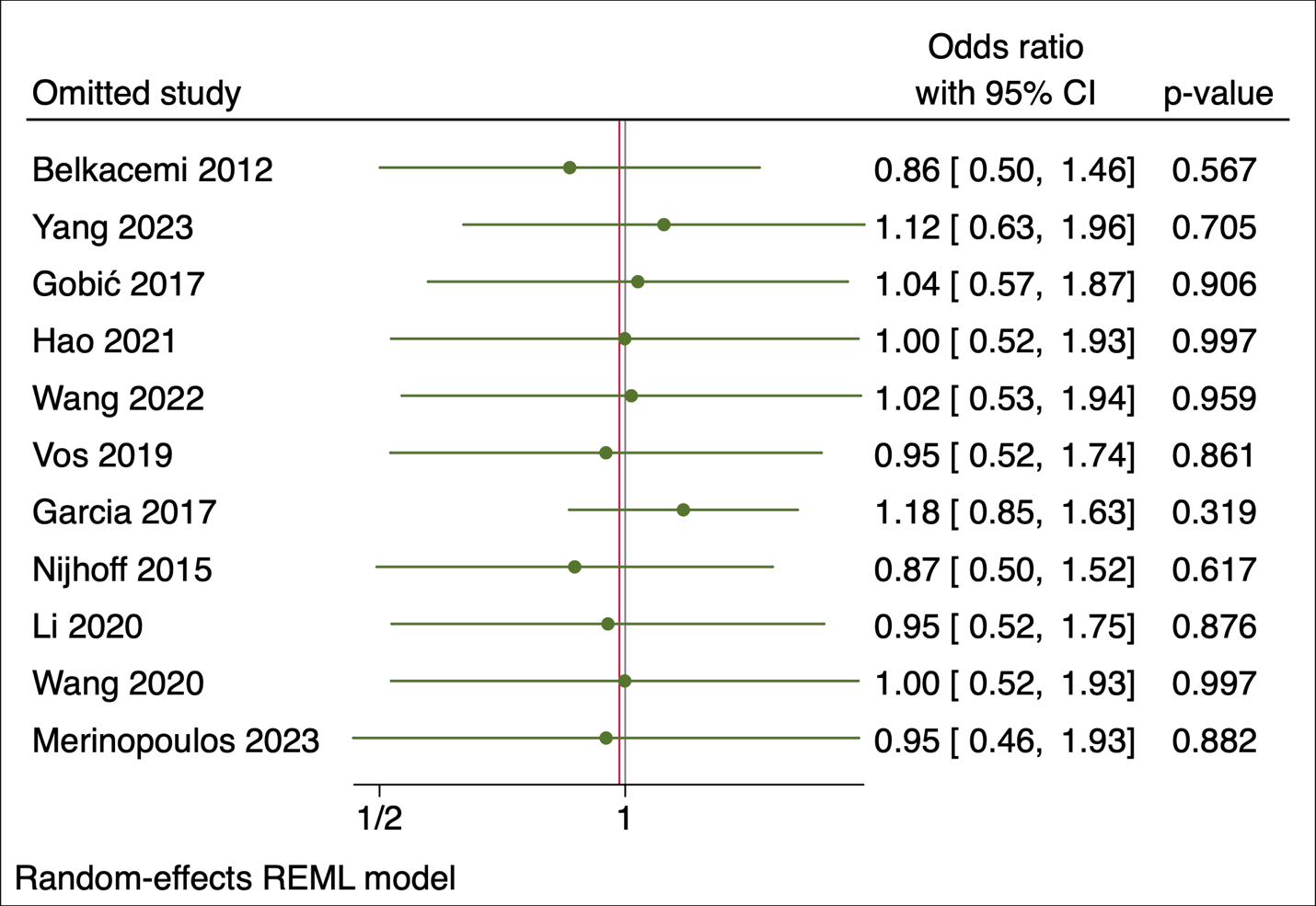


**Supplementary Figure 2:** Leave-one-out analysis of STEMI studies assessing MACE.


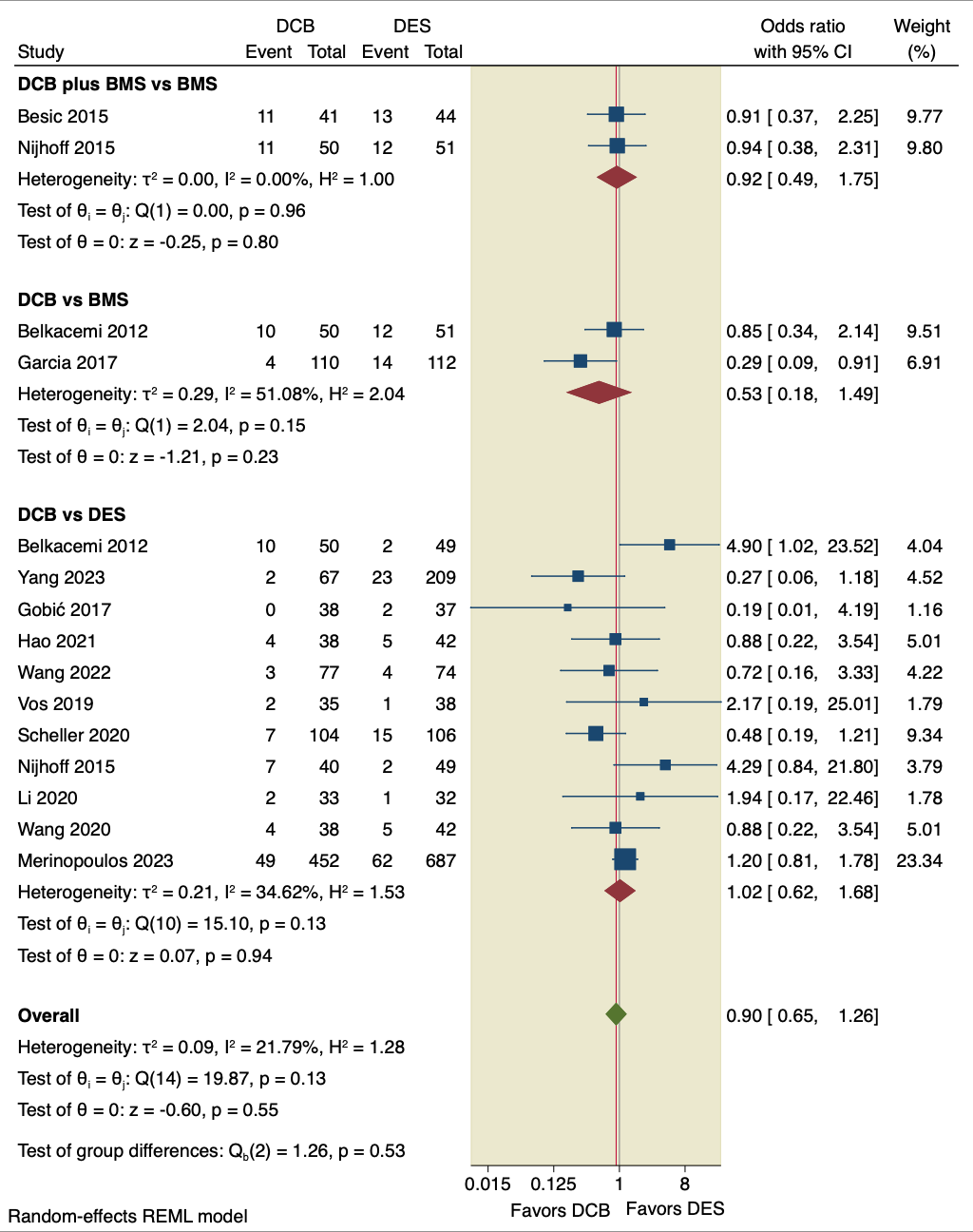


**Supplementary Figure 3:** Subgroup analysis of MACE based on the indication.


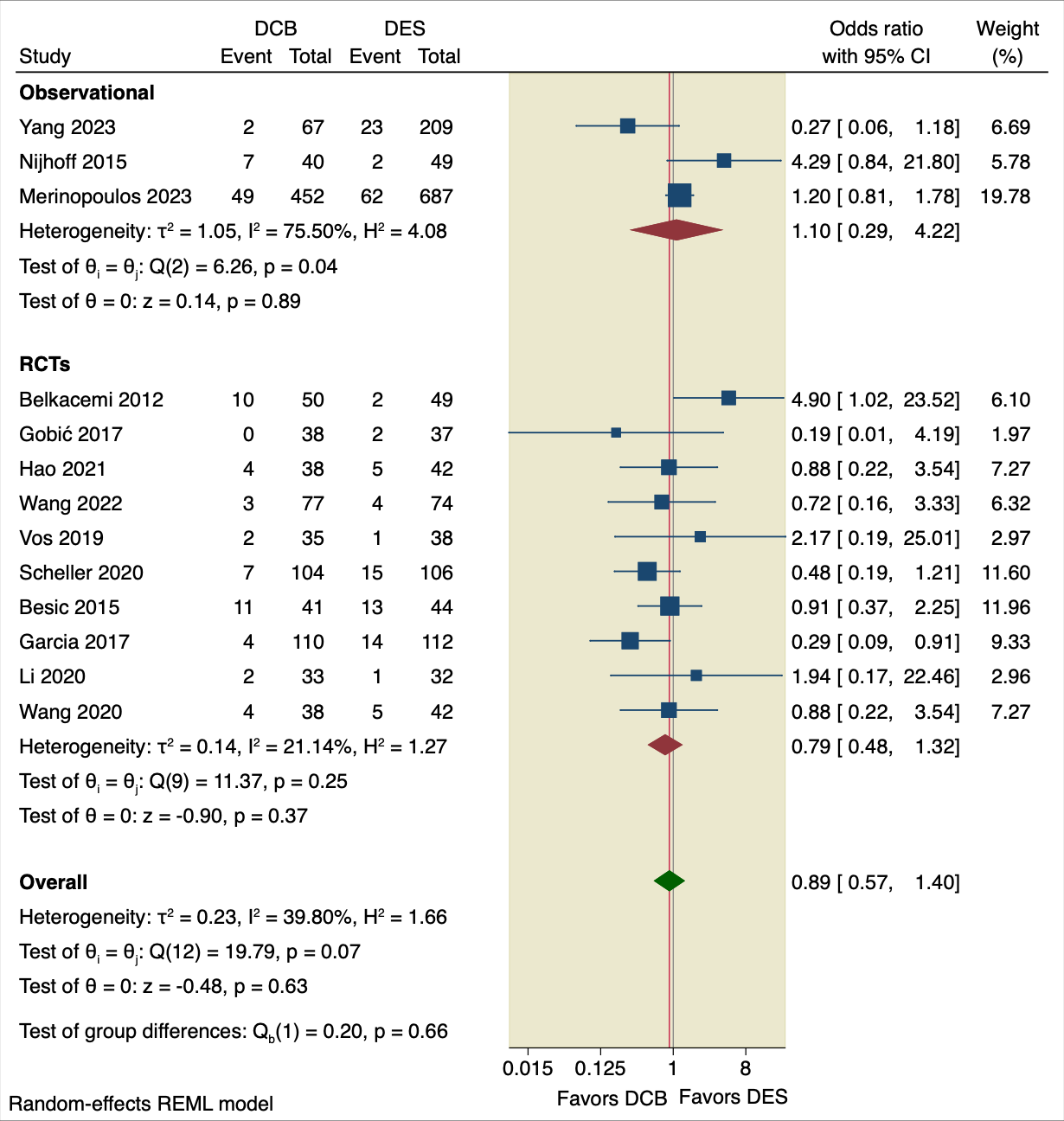


**Supplementary Figure 4:** Subgroup analysis of MACE based on study type.


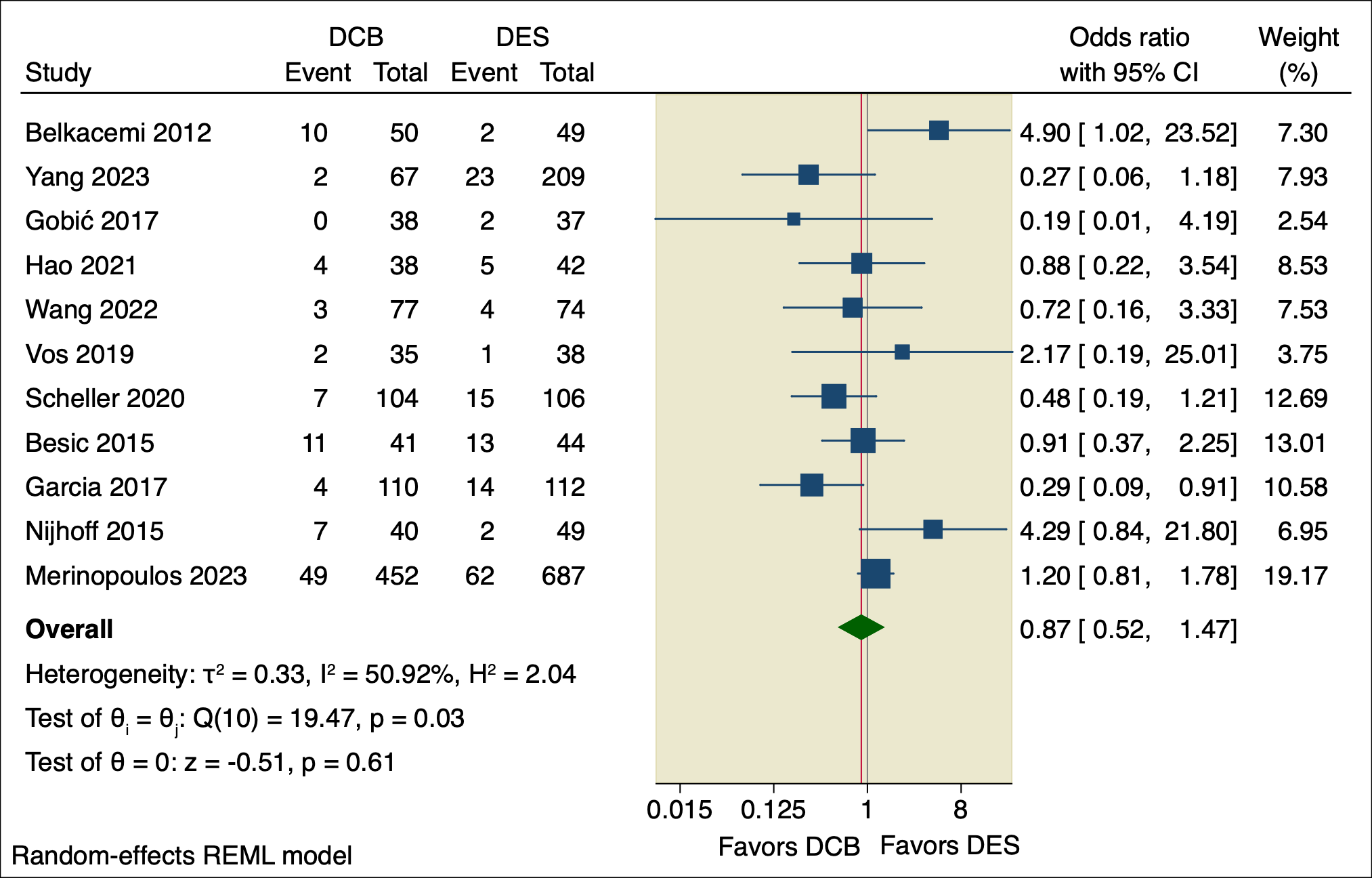


**Supplementary Figure 5:** Forest plot of MACE after excluding the Chinese studies.


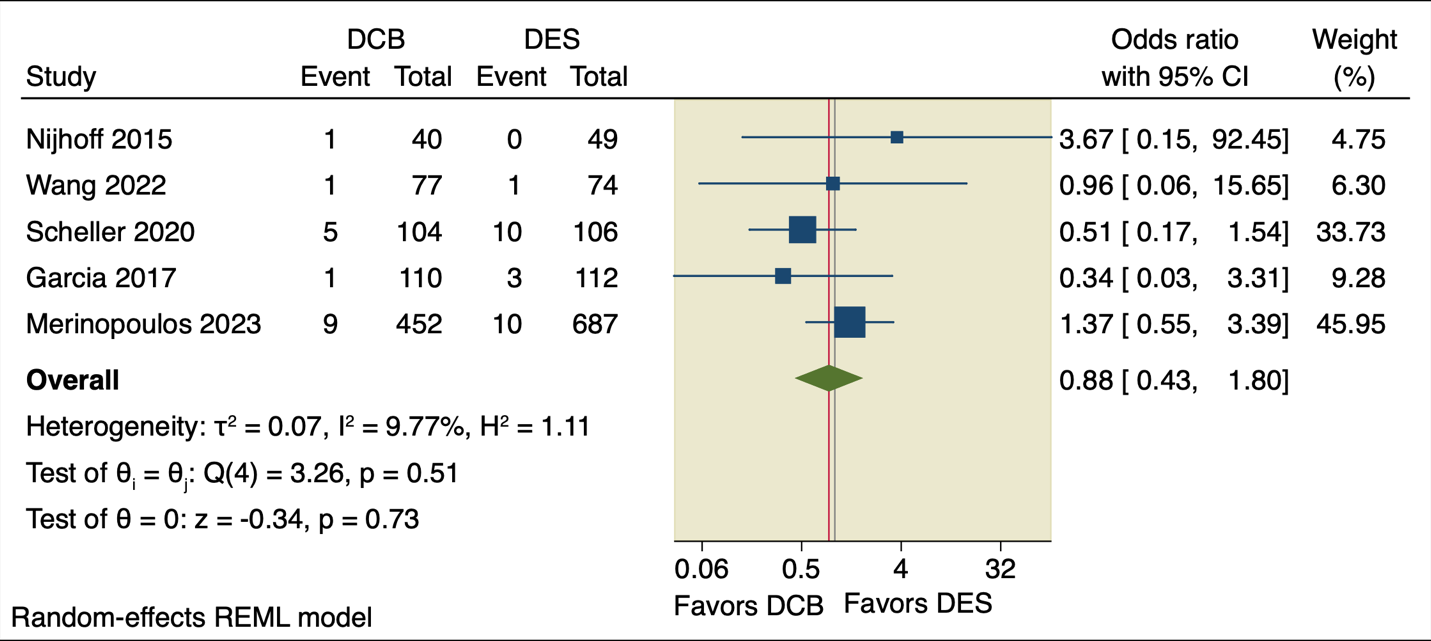


**Supplementary Figure 6:** Forest plot of all-cause mortality.


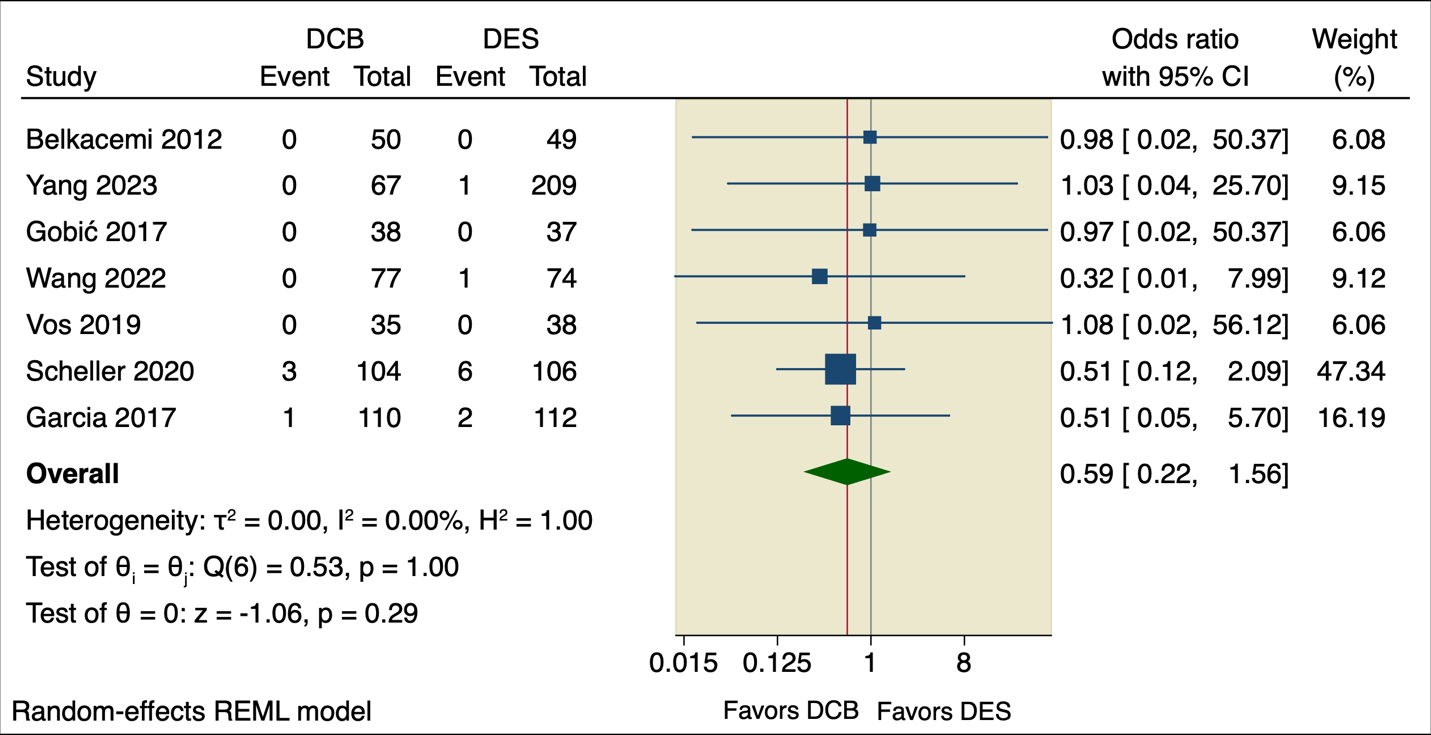


**Supplementary Figure 7:** Forest plot of cardiac mortality.


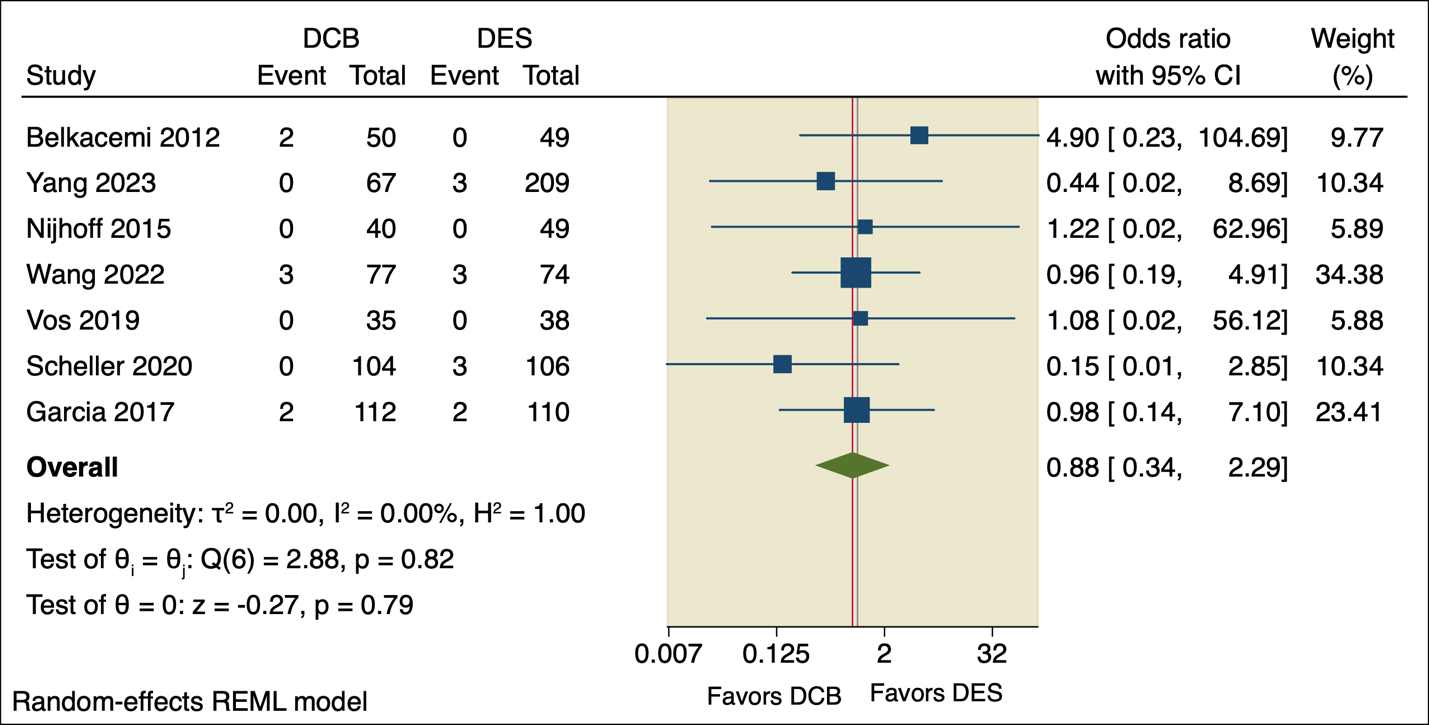


**Supplementary Figure 8:** Forest plot of MI.


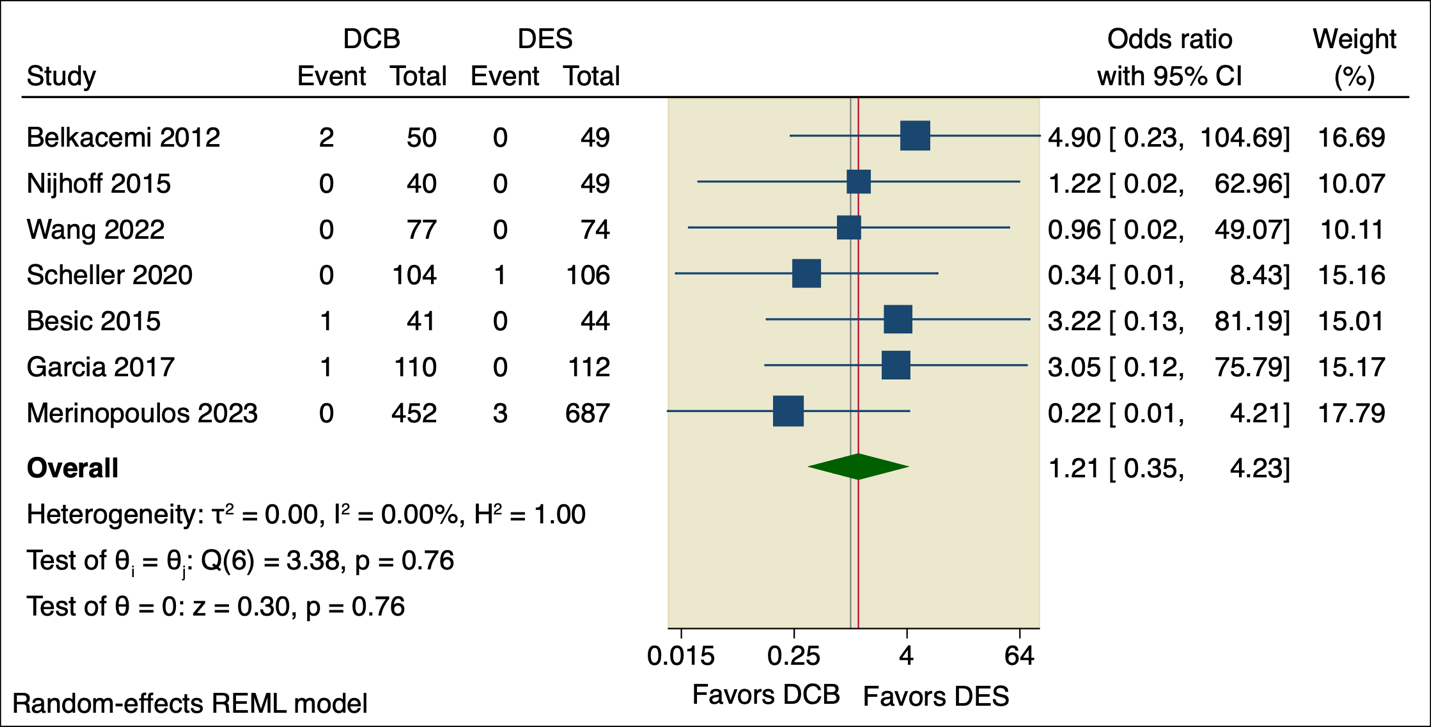


**Supplementary Figure 9:** Forest plot of stent thrombosis.


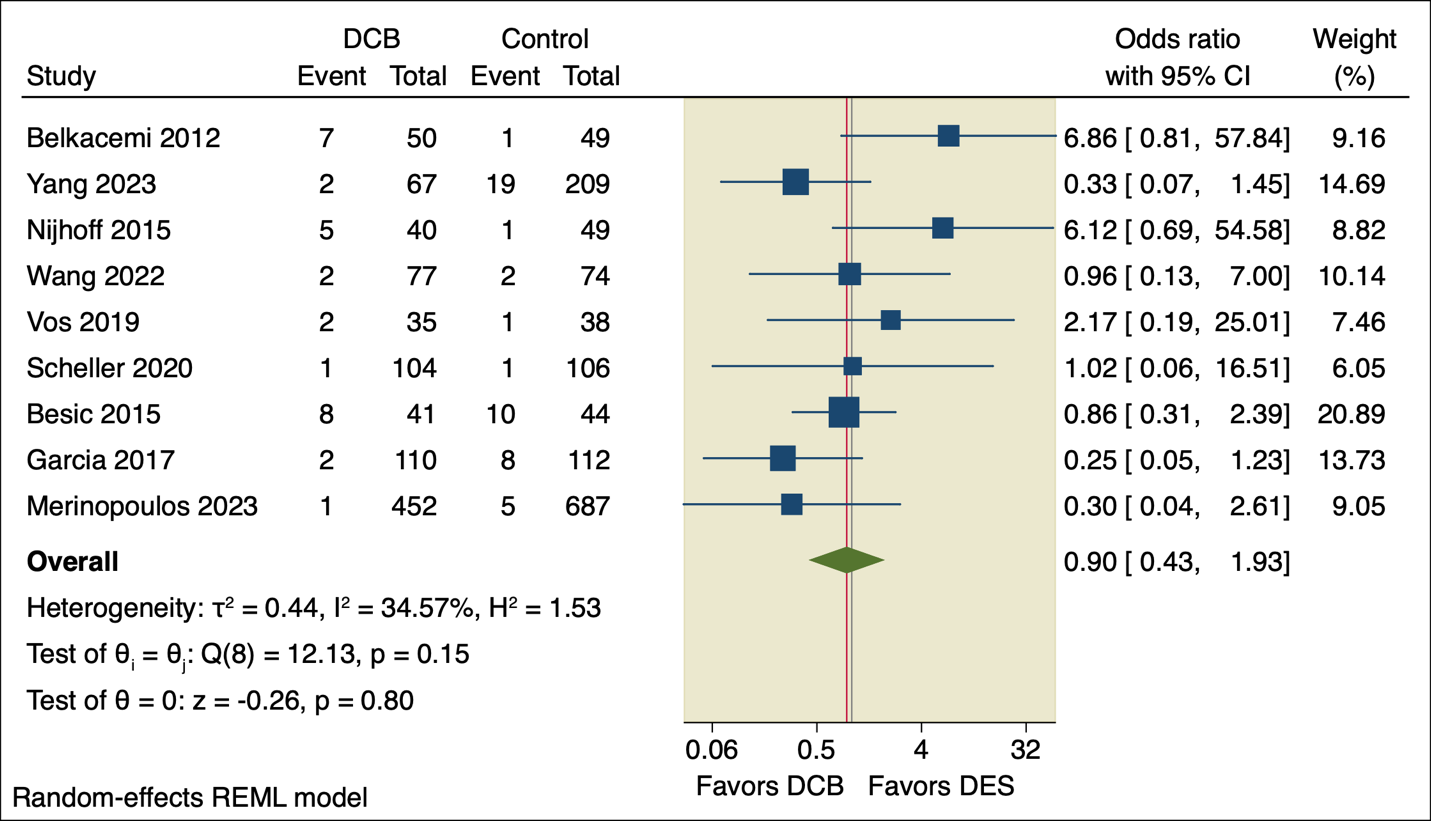


**Supplementary Figure 10:** Forest plot of TLR.


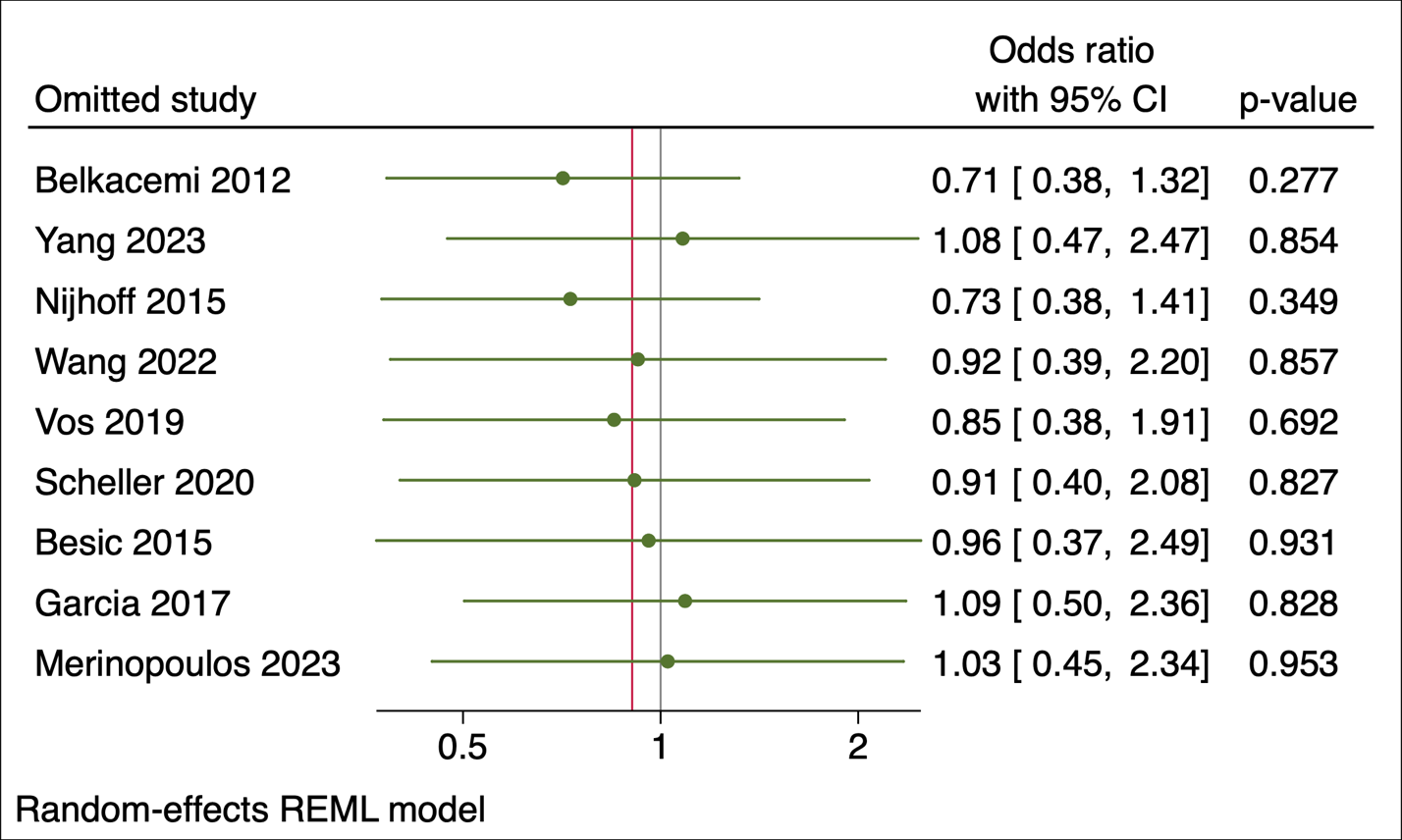


**Supplementary Figure 11:** Leave-one-out analysis of TLR.


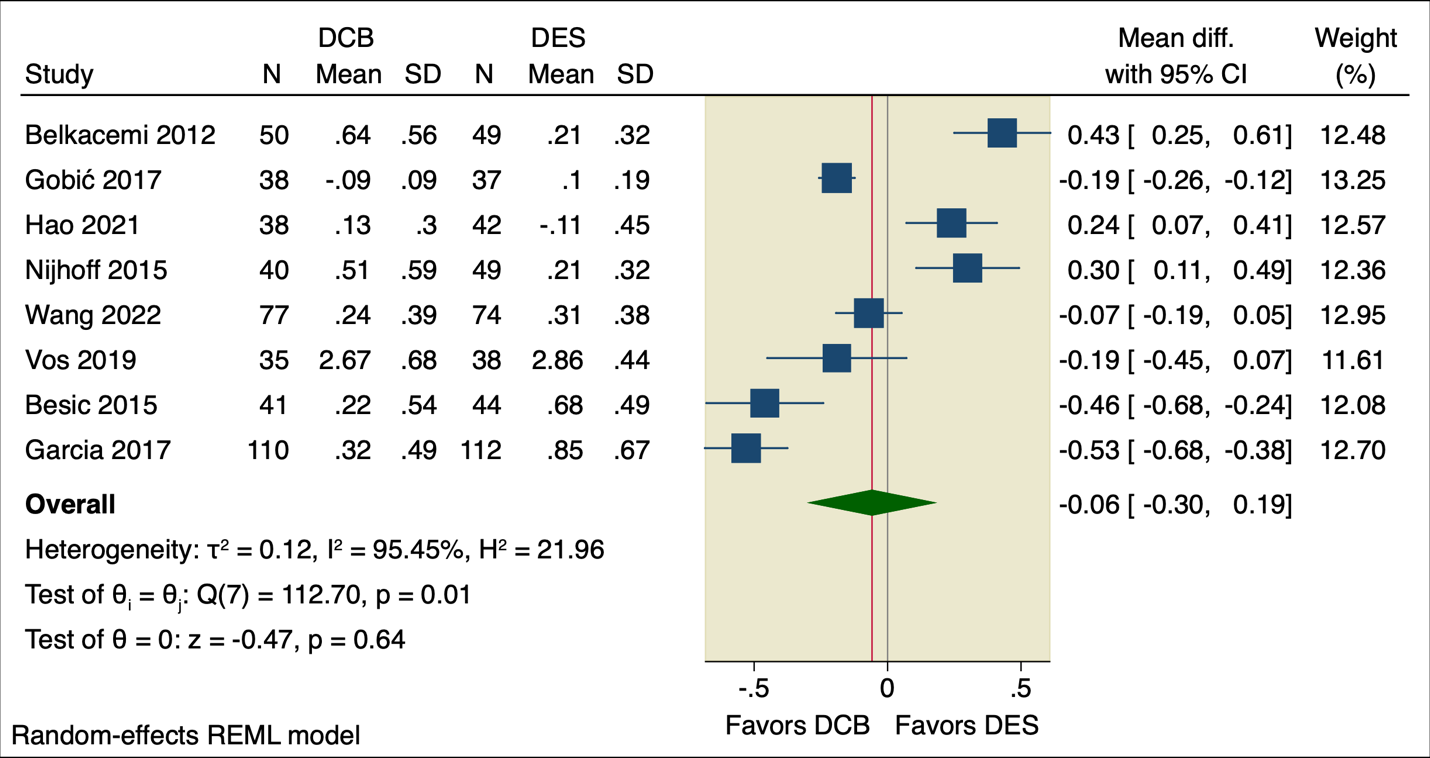


**Supplementary Figure 12:** Forest plot of LLL.


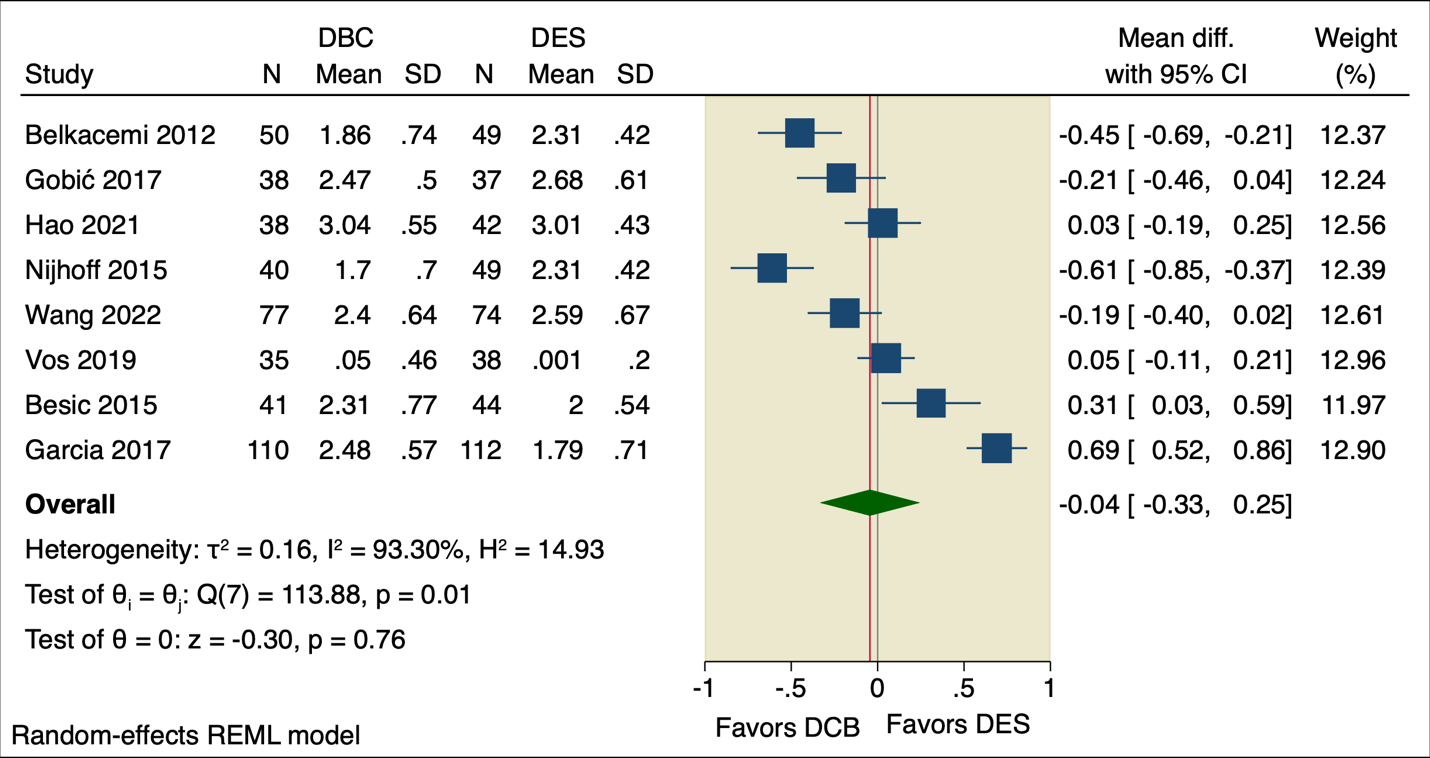


**Supplementary Figure 13:** Forest plot of MLD.


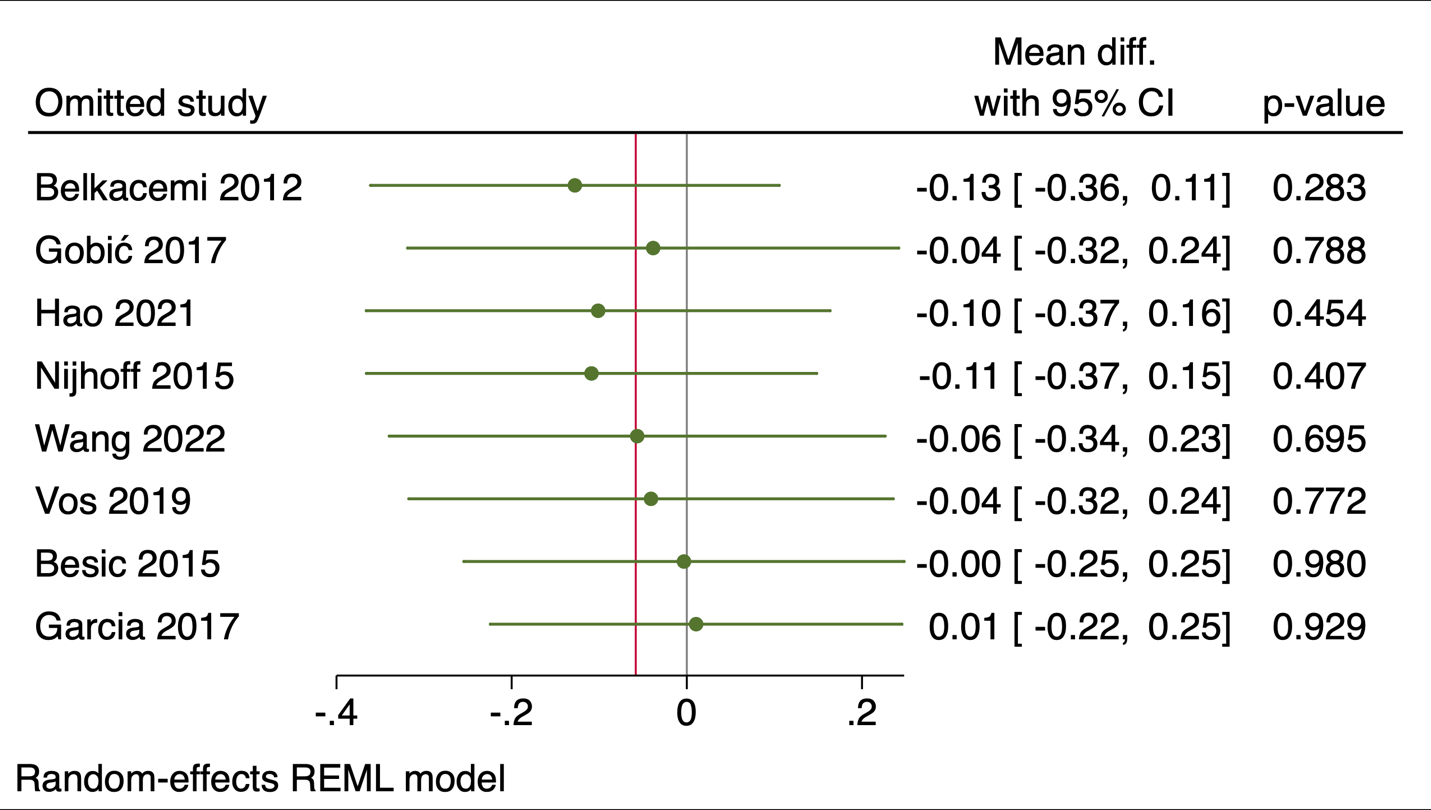


**Supplementary Figure 14:** Leave-one-out analysis of LLL.


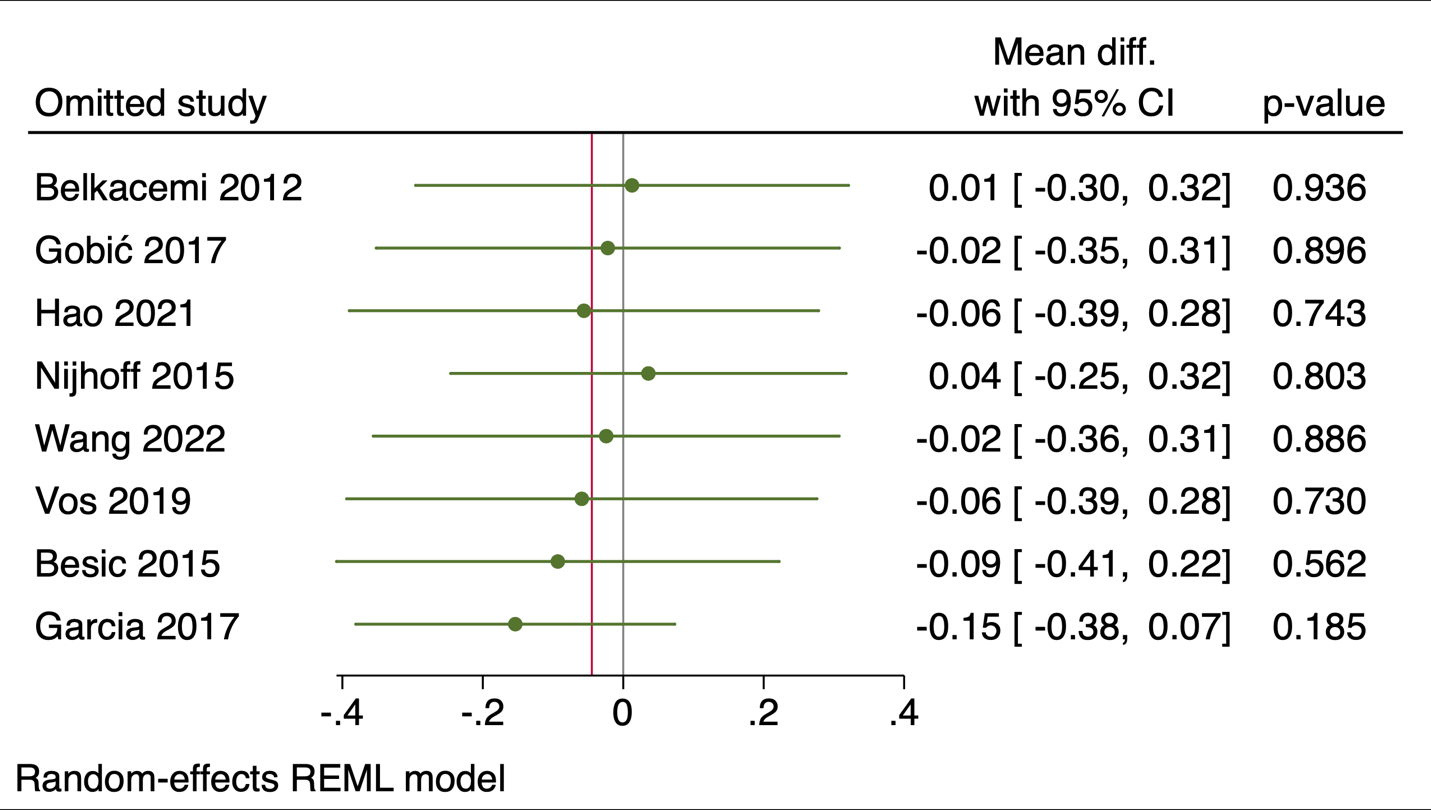


**Supplementary Figure 15:** Leave-one-out analysis of MLD.
