# Supplementary material for: Drug-coated balloons versus drug-eluting stents in patients with acute myocardial infarction undergoing percutaneous coronary intervention: an updated meta-analysis with trial sequential analysis
Source: BMC Cardiovasc Disord. 2023 Dec 8;23:605. doi: 10.1186/s12872-023-03633-w (PMC10709955; doi:10.1186/s12872-023-03633-w)
Supplement: Supplementary file 2 — Additional file 2. [file 12872_2023_3633_MOESM2_ESM.docx]

| Author, year | Description of outcome |
| --- | --- |
| MACE | |
| Belkacemi, 2012 | Major adverse cardiac events (MACE) were defined as a  hierarchical composition of death, any myocardial infarction  (MI), and target vessel revascularization (TVR). |
| Besic, 2015 | Major adverse cardiac events (MACE) were defined as a hierarchical composition of TLR, ST, and new acute coronary syndrome. |
| García-Touchard, 2017 | Major adverse cardiac events (MACE) were defined as a composite of death, non-fatal target vessel reinfection, or ischaemia-driven TVR. |
| Gobić, 2017 | Major adverse cardiac events (MACE) were followed during hospital stay: cardiovascular death, reinfection, target lesion revascularization and stent thrombosis. |
| Hao, 2021 | Major adverse cardiac events (MACE) events were defined as: cardiovascular death during follow-up, re-infarction or revascularization of target lesions. |
| Scheller, 2020 | Major adverse cardiovascular events (MACE) consisting of all-cause mortality, myocardial infarction, target lesion revascularisation, stroke, or PCI at other vessels. |
| Vos, 2019 | Major adverse cardiovascular events (MACE) consisting of Cardiac death, Recurrent MI, TLR and LVEF. |
| Wang, 2022 | Major adverse cardiac events (MACE) events were defined as: the composite of cardiac death, non-fatal myocardial infarction (MI), and target vessel revascularization (TVR) and stent thrombosis (ST). |
| Yang, 2023 | Major adverse cardiac events (MACE) events were defined as: cardiac death, target vessel myocardial infarction (MI), or target lesion revascularization (TLR). |
| Nijhoff, 2015 | Major adverse cardiac events (MACE) events were defined as: a hierarchical composition of death, any myocardial infarction and target vessel revascularization (TVR). |
| Liu, 2020 | Major adverse cardiac events (MACE) events were defined as: cardiac death, target vessel myocardial infarction (MI), or target lesion revascularization (TLR). |
| Wang, 2020 | Major adverse cardiac events (MACE) events were defined as: cardiac death, target vessel myocardial infarction (MI), or target lesion revascularization (TLR). |
| Merinopoulos 2023 | Major adverse cardiac events (MACE) events were defined as: cardiac death, target vessel myocardial infarction (MI), or target lesion revascularization (TLR). |

**Supplementary Table 1:** Definitions of MACE.
